# d-Mannose for Prevention of Recurrent Urinary Tract Infection Among Women: A Randomized Clinical Trial

**DOI:** 10.1001/jamainternmed.2024.0264

**Published:** 2024-04-08

**Authors:** Gail Hayward, Sam Mort, Alastair D. Hay, Michael Moore, Nicholas P. B. Thomas, Johanna Cook, Jared Robinson, Nicola Williams, Nicola Maeder, Rebecca Edeson, Marloes Franssen, Jenna Grabey, Margaret Glogowska, Yaling Yang, Julie Allen, Christopher C. Butler

**Affiliations:** 1 Nuffield Department of Primary Care Health Sciences, Oxford University, Oxford, England, United Kingdom; 2 Centre for Academic Primary Care, NIHR School for Primary Care Research, Bristol Medical School: Population Health Sciences, University of Bristol, Bristol, England, United Kingdom; 3 Primary Care Research Centre, Primary Care, Population Sciences and Medical Education, Faculty of Medicine, University of Southampton, Southampton, England, United Kingdom; 4 Windrush Medical Practice, Witney, England, United Kingdom; 5 NIHR Clinical Research Network Thames Valley and South Midlands, Oxford, England, United Kingdom; 6 Oxford Trauma and Emergency Care, Nuffield Department of Orthopaedics, Rheumatology and Musculoskeletal Sciences, Oxford University, England, United Kingdom

## Abstract

**Importance:**

Recurrent urinary tract infection (UTI) is a common debilitating condition in women, with limited prophylactic options. d-Mannose has shown promise in trials based in secondary care, but effectiveness in placebo-controlled studies and community settings has not been established.

**Objective:**

To determine whether d-mannose taken for 6 months reduces the proportion of women with recurrent UTI experiencing a medically attended UTI.

**Design, Setting, and Participants:**

This 2-group, double-blind randomized placebo-controlled trial took place across 99 primary care centers in the UK. Participants were recruited between March 28, 2019, and January 31, 2020, with 6 months of follow-up. Participants were female, 18 years or older, living in the community, and had evidence in their primary care record of consultations for at least 2 UTIs in the preceding 6 months or 3 UTIs in 12 months. Invitation to participate was made by their primary care center. A total of 7591 participants were approached, 830 responded, and 232 were ineligible or did not proceed to randomization. Statistical analysis was reported in December 2022.

**Intervention:**

Two grams daily of d-mannose powder or matched volume of placebo powder.

**Main Outcomes and Measures:**

The primary outcome measure was the proportion of women experiencing at least 1 further episode of clinically suspected UTI for which they contacted ambulatory care within 6 months of study entry. Secondary outcomes included symptom duration, antibiotic use, time to next medically attended UTI, number of suspected UTIs, and UTI-related hospital admissions.

**Results:**

Of 598 women eligible (mean [range] age, 58 [18-93] years), 303 were randomized to d-mannose (50.7%) and 295 to placebo (49.3%). Primary outcome data were available for 583 participants (97.5%). The proportion contacting ambulatory care with a clinically suspected UTI was 150 of 294 (51.0%) in the d-mannose group and 161 of 289 (55.7%) in the placebo group (risk difference, −5%; 95% CI, −13% to 3%; *P* = .26). Estimates were similar in per protocol analyses, imputation analyses, and preplanned subgroups. There were no statistically significant differences in any secondary outcome measures.

**Conclusions and Relevance:**

In this randomized clinical trial, daily d-mannose did not reduce the proportion of women with recurrent UTI in primary care who experienced a subsequent clinically suspected UTI. d-Mannose should not be recommended for prophylaxis in this patient group.

**Trial Registration:**

isrctn.org Identifier: ISRCTN13283516

## Key Points

**Question:**

Does daily d-mannose prevent recurrent urinary tract infection (UTI)?

**Findings:**

In this randomized clinical trial including 598 women with recurrent UTI recruited from primary care settings, the proportion experiencing a medically attended UTI was 51.0% in those taking daily d-mannose over 6 months and 55.7% in those taking placebo.

**Meaning:**

d-Mannose should not be recommended to prevent future episodes of medically attended UTI in women with recurrent UTI in primary care.

## Teaser

This randomized clinical trial seeks to determine whether d-mannose taken for 6 months reduces the proportion of women with recurrent urinary tract infections experiencing a medically attended urinary tract infection.

## Introduction

Urinary tract infection (UTI) is the most common bacterial infection affecting women presenting to primary care,^1^ with a lifetime risk of up to 50%.^2^ Recurrent UTI (rUTI), defined as experiencing 2 UTIs in 6 months or 3 in a year, has a considerable negative effect on quality of life, which extends beyond the unpleasant symptoms to distressing and disrupted sexual relationships, persistent and unmanageable pain, and systemic illness.^3,4^ In 2019, more than 400 million individuals had UTIs globally, and more than 200 000 people died of UTIs.^5^

The most common approach to prophylaxis of rUTI is daily antibiotic use. While effective during the period of prophylaxis, this increases risk of subsequent resistant UTIs and adverse effects.^6,7^ A recent open randomized trial in women who were referred to secondary care with rUTI found that methenamine hippurate was noninferior to prophylactic antibiotics,^8^ but there is limited evidence in primary care populations of effectiveness in comparison with placebo,^9^ and there are limited data on effectiveness or safety in older adults.^10^

d-Mannose is a food supplement found in small quantities in some fruits and vegetables^11^ that may offer an alternative to antibiotic prophylaxis in women who experience rUTI and, in turn, contribute to better antimicrobial stewardship in primary care. It is absorbed in the upper gastrointestinal tract and excreted in the urine.^12^ d-Mannose is a monosaccharide isomer of glucose that may inhibit bacterial adherence to uroepithelial cells by binding to a site on the tip of the fimbria^13^ and has shown benefit in animal models in preventing UTIs.^14^ Although a recent Cochrane systematic review concluded that there was insufficient evidence to support d-mannose for UTI prophylaxis,^15^ an open randomized trial that compared d-mannose, antibiotic prophylaxis, and usual care found evidence of benefit.^16^ The high costs (at least £23 a month in the UK [US $29]) add weight to the need to establish whether family physicians should advise patients to buy d-mannose, since it is not available on prescription. In this randomized double-blind placebo-controlled study, we compared the clinical effectiveness of d-mannose and placebo powder to prevent UTI in women with rUTI in primary care.

## Methods

### Study Design and Oversight

We assessed the effectiveness of d-mannose in a multicenter, primary care, 2-group, double-blind, placebo-controlled randomized clinical trial in the UK. The South West–Central Bristol Research Ethics Committee reviewed and approved the trial protocol (Supplement 1). Online informed consent was obtained from all participants. An independent trial steering committee provided trial oversight. Reporting is in line with the Consolidated Standards of Reporting Trials (CONSORT) reporting guidelines.^17^ The full trial protocol is published.^18^

### Participants

Women were identified either by responding to an advertisement in their surgery, on presentation to their primary care clinician, or by participating primary care centers through records search followed by a written invitation to participate. We recruited from 99 primary care centers based in 10 of 15 regions of England and 4 of 7 health boards in Wales. Practice locations spanned the full range of the Index of Multiple Deprivation deciles, with 40% based in deciles 1 to 5. The trial team or recruiting site established eligibility and took informed consent via a conversation either face to face or by telephone using an online form. Women were eligible if they were 18 years or older, willing and able to give informed consent for participation and comply with study procedures, and had presented to ambulatory care with symptoms consistent with a UTI and/or resulting in a UTI-specific antibiotic prescription 3 or more times in the past year or 2 or more times in the past 6 months. Women were ineligible if they were pregnant, lactating, or planning pregnancy during the study; had formal diagnosis of interstitial cystitis or overactive bladder syndrome; were a nursing home resident; catheterized, including intermittent self-catheterization; using Uromune (Inmunotek); or had previously participated in this study or had participated in a research study involving an investigational medicinal product in the past 12 weeks. Women were also ineligible if they had started prophylactic antibiotics in the past 3 months and were unwilling to discontinue or intended to start them during the next 6 months or were currently using d-mannose and unwilling to discontinue it.

### Randomization and Masking

Eligible, consenting participants were randomized in equal allocation between d-mannose and placebo using a secure, web-based randomization system (Sortition). Block randomization was implemented with varying block sizes. Randomization was stratified by primary care center. Participants, clinicians, and members of the trial team responsible for recruitment/follow-up/monitoring of participants were blinded to group assignment. To conceal allocation, bottles of d-mannose and fructose (placebo) looked identical and were randomly assigned a number that was used in the randomization process.

### Procedures

Participants were randomized to either take a daily scoop amounting to approximately 2 g of d-mannose powder or a similar, daily scoop of fructose powder. Both study products were white powders with a similar sweet taste. Fructose is absorbed in the small intestine and almost completely metabolized by the liver.^19^ Participants continued to take the study product when symptomatic and when taking antibiotics. The investigational medicinal product and placebo were purchased from Tiofarma, which had no role in the study design or conduct. Participants were sent packs of powder every 2 months. Participants were asked to complete a daily symptom diary covering severity of UTI symptoms, use of over-the-counter medicines or antibiotics, health care contacts, and the EuroQol EQ-5D-5L instrument (on days 1, 3, and 5 only) until their symptoms resolved. Some women had continuous, mild symptoms of UTI, and if this was the case, they were asked to complete the symptom diary if they experienced a flare of their symptoms. A weekly questionnaire, completed by weblink from text message, email, or by telephone, captured adherence and symptomatic episodes where a symptom diary had not been completed. Participants who responded to fewer than 3 of 4 weekly questionnaires received in addition a monthly telephone call. At the end of 6 months, a final questionnaire covering adherence, the EQ-5D-5L, and whether participants thought that they had been taking d-mannose or placebo was completed. A primary care medical records review was performed to collect details of presentations to ambulatory care (all clinical contacts are notified to the primary care practitioner in the UK) with symptoms consistent with a UTI and clinically recorded as a UTI during the 6 months of study participation (primary outcome), details of urine cultures performed, antibiotic prescriptions, and hospitalizations.

### Outcomes

The primary outcome was the proportion of women experiencing at least 1 further episode of clinically suspected UTI for which they contacted ambulatory care (including primary care, emergency departments, hospitals, ambulance services, and out-of-hours primary care) within 6 months of randomization established via primary care record review. This outcome was chosen after discussion with women with lived experience of rUTI as the most important indicator of symptom burden, which prophylaxis would aim to avoid. In primary care, microbiological confirmation is not generally relied on for guiding initial antibiotic prescribing and in up to one-third of cases will be inconclusive.^20^

Secondary outcomes included number of days of moderately bad (or worse) symptoms of UTI recorded in symptom diaries, time to next consultation with a clinically suspected UTI, number of clinically suspected UTIs, number of microbiologically proven UTIs, number and consumption of antibiotic courses for UTI, defined daily dose and total milligram by antibiotic type, proportion of women with resistant uropathogens cultured during an episode of acute infection, and hospital admissions related to UTI. We documented serious adverse events, excluding hospitalizations for elective procedures. Microbiological outcomes were based on urine samples sent by primary care clinicians for routine care, available on clinical record review. Outcomes including quality of life, health care utilization and acceptability, microbiological analyses using samples provided to our central laboratory, and process evaluation will be reported elsewhere.

### Statistical Analysis

A 2016 study evaluating prophylactic treatment for rUTIs in a similar population found that 26.6% of women in the control group experienced a UTI within 6 months.^21^ The patient advisory panel for the present study suggested that to commit to daily use of a prophylactic regime they would expect evidence of at least a 50% reduction in the chance of a further UTI during the period of prophylaxis. To detect this with 90% power and an α of .05, we required 203 participants in each group, equating to 508 participants in total with allowance for 20% loss to follow-up. This sample size was also adequate to power the key secondary outcome (the number of UTIs experienced over 6 months) and detect a relative incidence rate of 0.5 between the treatment and placebo groups, assuming a base rate of 0.36 as estimated by Maki et al.^21^ Since the withdrawal rate was higher than our anticipated 20% in interim blinded data review, the sample size was increased to 598 to allow for additional withdrawals on November 7, 2019.

The primary analysis population was defined as all eligible participants randomized to d-mannose or placebo. Analysis was according to group allocation regardless of protocol deviation.

The proportion of women experiencing at least 1 further episode of a clinically suspected UTI was analyzed using a log-Poisson generalized linear mixed-effects model with robust standard errors. The model included randomized group as a fixed effect and adjusted for site as a random effect, included in the model as a random coefficient. The sensitivity analyses and the following secondary outcomes were analyzed in the same way: total number of clinically suspected UTIs, number of microbiologically proven UTIs, number of prescribed antibiotic courses for UTI, report of consumption of antibiotics, count of consumption of antibiotics, incidence of antibiotic-resistant UTIs, count of antibiotic-resistant UTIs, hospital admissions related to UTIs, and count of hospital admissions related to UTIs. For imputation sensitivity analyses, we imputed missing values as positive (assuming everyone with missing data experienced at least 1 further episode of a clinically suspected UTI) and negative (assuming everyone with missing data did not experience at least 1 further episode). Subgroup analyses were conducted in the same way as for the primary analysis but included an additional fixed effect for the categorical subgroup variable and an interaction term for the subgroup variable and randomized group. The secondary outcome of defined daily dose was analyzed using a linear mixed-effects model. Time to next consultation with a clinically suspected UTI was analyzed using a mixed-effects Cox proportional hazard model. Both models included randomized group as a fixed effect and adjusted for site as a random effect. The secondary outcomes of number of days of moderately bad (or worse) symptoms of UTI and number of days prescribed antibiotics for UTI were highly skewed and were analyzed using a quantile regression adjusted for randomized group. See the statistical analysis plan in Supplement 2 for additional information.

Statistical analysis was reported in December 2022. Stata, version 16.1 (StataCorp), was used for analyses. The level of statistical significance was set at *P* = .05, and tests were 2-sided.

## Results

### Population

**Figure 1.**
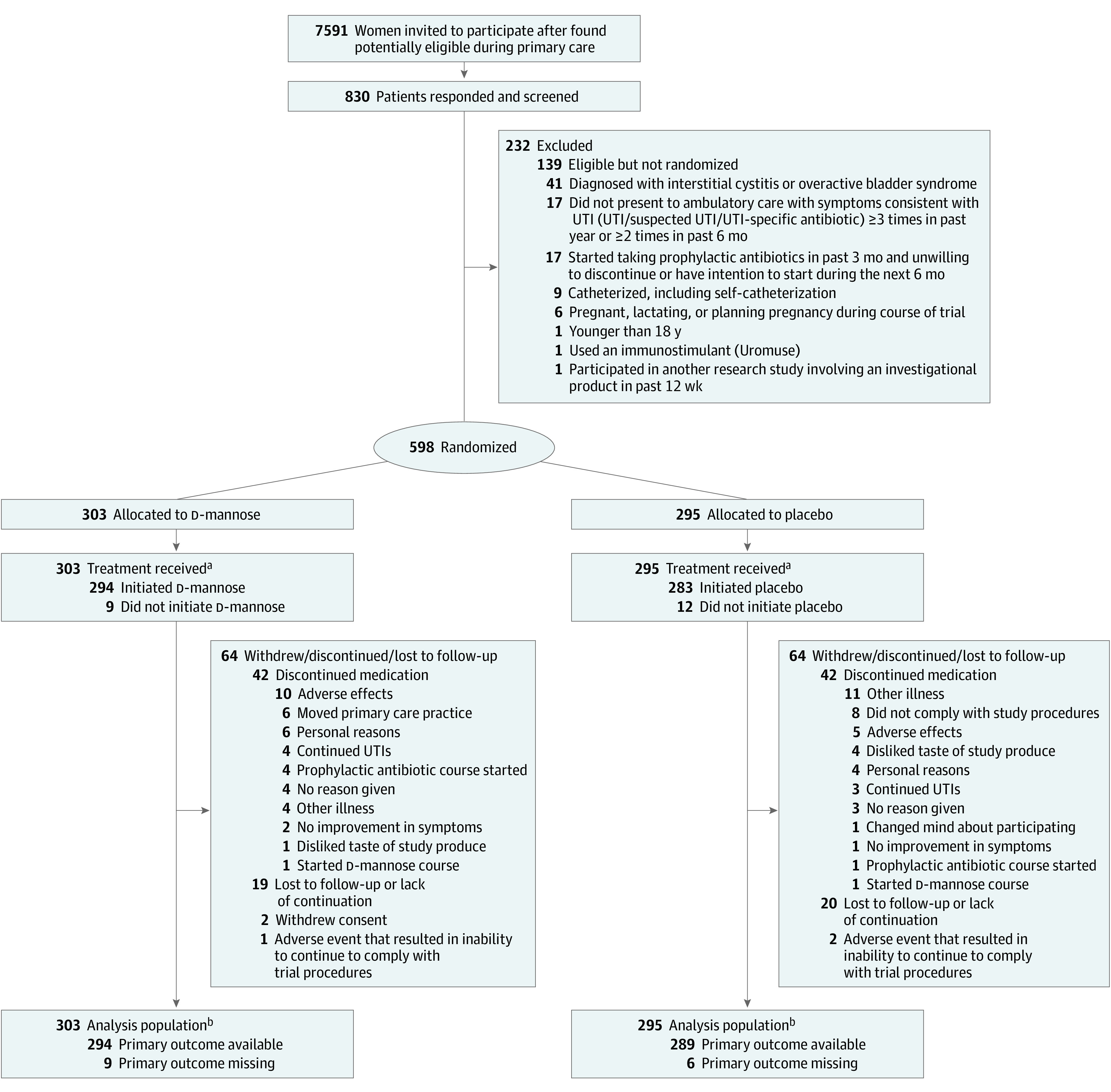
Patient Flowchart UTI indicates urinary tract infection. ^a^Self-reported taking at least 1 dose of the study medication during the study period. ^b^Participants were only excluded from the primary analysis population if primary outcome data were unavailable.

Between March 28, 2019, and January 31, 2020, 598 women were randomized to d-mannose (n = 303) or placebo (n = 295). The mean (range) age of participants was 58.6 (19.0-91.9) years in the d-mannose group and 57.3 (18.2-93.5) years in the placebo group. Baseline characteristics were similar between the comparison groups (Table 1 and eTable 1 in Supplement 3). Withdrawals totaled 69 women in the d-mannose group and 65 in the placebo group. Primary outcome data were available for 294 (97.0%) women in the d-mannose group and 289 (98.0%) in the placebo group (Figure 1).

**Table 1.** Baseline Characteristics by Participant Response in Randomized Group

Characteristic	No. (%)		
	d-Mannose (n = 303)	Placebo (n = 295)	Overall (N = 598)
Age, y			
Mean (SD)	58.6 (17.08)	57.3 (19.08)	58.0 (18.09)
Median (IQR) [range]	61.4 (48.3-72.5) [19.0-91.9]	61.3 (43.4-72.9) [18.2-93.5]	61.3 (46.5-72.8) [18.2-93.5]
Missing	0	0	0
Have you gone through the menopause?			
Yes	194 (64.0)	182 (62.1)	376 (63.1)
No	86 (28.4)	97 (33.1)	183 (30.7)
Do not know	23 (7.6)	14 (4.8)	37 (6.2)
Missing	0	2	2
If yes, do you use hormonal treatment for your menopause?^a^			
Tablet	15 (7.7)	15 (8.2)	30 (8.0)
Patch	2 (1.0)	3 (1.6)	5 (1.3)
Vaginal	31 (16.0)	27 (14.8)	58 (15.4)
None	136 (70.1)	131 (72.0)	267 (71.0)
Missing	13	7	20
Are you using any of the following contraceptives?^a^			
Combined pill	14 (4.6)	22 (7.5)	36 (6.0)
Progesterone-only pill	16 (5.3)	8 (2.7)	24 (4.0)
Coil, copper	5 (1.7)	2 (0.7)	7 (1.2)
Coil, hormonal	23 (7.6)	17 (5.8)	40 (6.7)
Implant	2 (0.7)	8 (2.7)	10 (1.7)
None of the above	225 (74.3)	223 (75.6)	448 (74.9)
Missing	21	16	37
Do you experience episodes of urine incontinence (leaking)?			
Yes	128 (42.4)	112 (38.1)	240 (40.3)
No	174 (57.6)	182 (61.9)	356 (59.7)
Missing	1	1	2
If yes, does this happen more than once a month?			
Yes	96 (75.0)	90 (81.8)	186 (78.2)
No	32 (25.0)	20 (18.2)	52 (21.8)
Missing	0	2	2
How many UTI episodes have you had in the past 12 mo (self-reported)?			
Mean (SD)	4.4 (2.07)	4.8 (2.20)	4.6 (2.14)
Median (IQR) [range]	4.0 (3.0-6.0) [1.0-10.0]	4.0 (3.0-6.0) [1.0-10.0]	4.0 (3.0-6.0) [1.0-10.0]
1	2 (0.7)	4 (1.4)	8 (1.2)
2	37 (12.4)	17 (5.8)	61 (9.0)
3	86 (28.8)	79 (27.1)	186 (27.3)
4	66 (22.1)	69 (23.6)	153 (22.5)
5	32 (10.7)	41 (14.0)	89 (12.6)
6	39 (13.0)	30 (10.3)	79 (11.6)
7	11 (3.7)	11 (3.8)	28 (4.1)
8	7 (2.3)	16 (5.5)	28 (4.1)
9	1 (0.3)	2 (0.7)	3 (0.4)
10	18 (6.0)	23 (7.9)	49 (7.2)
Missing	4	3	7
How many of those were in the past 6 mo?			
Mean (SD)	2.8 (1.58)	2.9 (1.53)	2.8 (1.56)
Median (IQR) [range]	2.0 (2.0-3.0) [1.0-10.0]	3.0 (2.0-4.0) [1.0-10.0]	3.0 (2.0-3.0) [1.0-10.0]
Missing	7	5	12
How long did the episode last (in days)?^b^			
Mean (SD)	7.5 (6.30)	8.2 (8.74)	7.9 (7.61)
Median (IQR) [range]	6.0 (4.3-8.8) [1.0-65.0]	6.0 (4.3-8.0) [1.3-90.0]	6.0 (4.3-8.5) [1.0-90.0]
Missing	15	13	28
No. of contacts with a physician, nurse, or health care assistant because of previous UTI episode symptoms^c^			
0	14 (4.6)	15 (5.1)	29 (4.8)
1	35 (11.6)	37 (12.5)	72 (12.0)
2	86 (28.4)	63 (21.4)	149 (24.9)
≥3	168 (55.4)	180 (61.0)	348 (58.2)
Missing	0	0	0
Did you take anything to help with your symptoms?^d^			
Yes	277 (91.4)	265 (89.8)	542 (90.6)
Paracetamol	144 (52.0)	159 (60.0)	NA
Ibuprofen	70 (25.3)	57 (21.5)	NA
Cranberry juice	108 (39.0)	94 (35.5)	NA
Cystitis relief sachets^e^	102 (36.8)	89 (33.6)	NA
Other	178 (64.3)	178 (67.2)	NA
No	26 (8.6)	30 (10.2)	56 (9.4)
Missing	0	0	0
Are you currently experiencing symptoms of a UTI?			
Yes	67 (22.2)	75 (25.6)	142 (23.9)
No	235 (77.8)	218 (74.4)	453 (76.1)
Missing	1	2	3
How often do you generally have sexual intercourse?			
Not in the past year	117 (39.1)	115 (39.1)	232 (39.1)
A few times per year to monthly	38 (12.7)	39 (13.3)	77 (13.0)
A few times per month to weekly	77 (25.8)	67 (22.8)	144 (24.3)
2-3 Times per week	36 (12.0)	46 (15.6)	82 (13.8)
≥4 Times per week	12 (4.0)	14 (4.8)	26 (4.4)
Prefer not to say	19 (6.4)	13 (4.4)	32 (5.4)
Missing	4	1	5

Abbreviations: NA, not applicable; UTI, urinary tract infection.

^a^ Not mutually exclusive.

^b^ On average for up to the previous 3 episodes.

^c^ Total number of self-reported health care contacts for up to 3 previous UTI episodes.

^d^ Yes if for any of the previous 3 UTI episodes; no if for none of the previous 3 episodes.

^e^ Over-the-counter sachets containing sodium citrate or potassium citrate.

### Primary Outcome

Of 294 women in the d-mannose group and 289 in the placebo group, 150 (51.0%) and 161 (55.7%) had a further episode of clinically suspected UTI, respectively, for which they contacted ambulatory care (relative risk [RR], 0.92; 95% CI, 0.80-1.05; *P* = .22; and unadjusted risk difference, −5%; 95% CI, −13% to 3%; *P* = .26; Table 2). The proportion was similar in sensitivity analyses, including per protocol analysis (average adherence reported at 4 days a week or 3 days a week), when adjusting the model in the primary analysis for factors that predict missingness (taking anything to help with symptoms for previous UTI episode at baseline), and when imputing missing values for the primary outcome as positive or negative (eTable 2 in Supplement 3). The proportion was also similar in subgroup analyses comparing women with a history of more or less frequent UTIs and premenopausal vs postmenopausal women (eTable 3 in Supplement 3). Finally, a complier average causal effect analysis found no statistically significant influence of compliance on treatment effect (eTable 4 in Supplement 3).

**Table 2.** Frequency of Women Experiencing at Least 1 Further Episode of a Clinically Suspected Urinary Tract Infection (UTI) for Which They Contacted Ambulatory Care by Randomized Group

≥1 Further episode of a clinically suspected UTI	No. (%)		*P* value
	d-Mannose (n = 303)	Placebo (n = 295)	
Yes	150 (51.0)	161 (55.7)	NA
No	144 (49.0)	128 (44.3)	NA
Missing	9	6	NA
Unadjusted risk difference (95% CI)	−0.05 (−0.13 to 0.03)		.26
Adjusted relative risk (95% CI)^a^	0.92 (0.80 to 1.05)		.22

Abbreviation: NA, not applicable.

^a^ Log-Poisson generalized linear mixed-effects model with robust standard errors of the proportion of women experiencing at least 1 further episode of a clinically suspected UTI modeled against randomized arm as a fixed effect and site as a random effect. The level of statistical significance is *P* = .05.

### Secondary Outcomes

**Figure 2.**
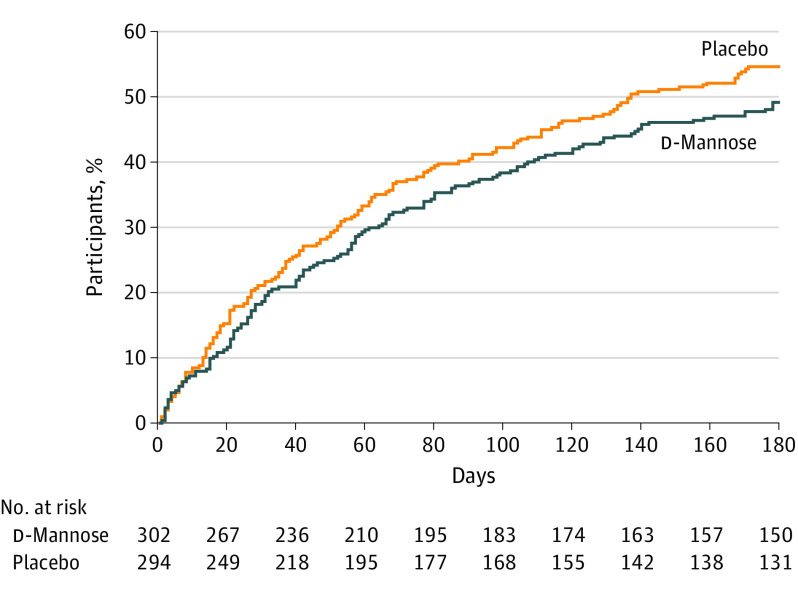
Time to Next Consultation With Clinically Suspected Urinary Tract Infection

There were no differences between d-mannose and placebo groups in the duration of moderately bad or worse symptoms of UTI recorded in symptom diaries (adjusted median difference: daily, 0.00; 95% CI, −0.36 to 0.36; *P* > .99; and weekly, 0.00; 95% CI, −0.37 to 0.37; *P* > .99) or the time to next consultation with a clinically suspected UTI (Table 3 and Figure 2). There were also no differences in the number of clinically suspected UTIs or microbiologically proven UTIs (Table 3, with more detail in eTable 5 in Supplement 3).

**Table 3.** Secondary Outcome Measures by Randomized Group

Outcome	d-Mannose (n = 303)	Placebo (n = 295)	*P* value
No. of days of moderately bad (or worse) symptoms of UTI			
Adjusted median difference (95% CI)^a^	0.00 (−0.36 to 0.36)		>.99
Median (IQR) [range]	0.0 (0.0 to 2.0) [0.0 to 18.0]	0.0 (0.0 to 2.0) [0.0 to 148.0]	NA
Mean (SD)	1.7 (3.13)	3.2 (12.15)	NA
Participants reporting a single day of moderately bad (or worse) symptoms, No. (%)	104 (43.3)	99 (43.2)	NA
Median (IQR) for those reporting a single day	3.0 (1.0 to 5.0)	3.0 (1.0 to 6.0)	NA
Missing	63	66	NA
Weekly symptom burden of UTI			
Adjusted median difference (95% CI)^a^	0.00 (−0.37 to 0.37)		>.99
Median (IQR) [range]	0.0 (0.0 to 1.0) [0.0 to 4.0]	0.0 (0.0 to 1.0) [0.0 to 5.0]	NA
Mean (SD)	0.5 (0.80)	0.6 (0.86)	NA
Missing	85	87	NA
Time to next consultation (days) with a clinically suspected UTI			
Adjusted hazard ratio (95% CI)^b^	0.86 (0.69 to 1.08)		.20
Time at risk, d	36 263	33 337	NA
Incidence rate (per 1000 d at risk)	4.11	4.80	NA
Missing^c^	1	1	NA
No. of clinically suspected UTIs			
Adjusted incidence rate ratio (95% CI)^d^	0.88 (0.72 to 1.08)		.21
Mean (SD)	0.9 (1.19)	1.0 (1.28)	NA
Median (IQR) [range]	1.0 (0.0 to 1.0) [0.0 to 6.0]	1.0 (0.0 to 2.0) [0.0 to 8.0]	NA
Missing	9	6	NA
No. of microbiologically proven UTIs			
Adjusted incidence rate ratio (95% CI)^d^	0.97 (0.63 to 1.48)		.88
Mean (SD)	0.4 (0.87)	0.4 (0.78)	NA
Median (IQR) [range]	0.0 (0.0 to 0.0) [0.0 to 4.0]	0.0 (0.0 to 1.0) [0.0 to 4.0]	NA
Missing	54	51	NA
No. of prescribed antibiotic courses for UTI			
Adjusted incidence rate ratio (95% CI)^d^	0.88 (0.69 to 1.12)		.29
Mean (SD)	0.9 (1.32)	1.1 (1.53)	NA
Median (IQR) [range]	0.0 (0.0 to 1.0) [0.0 to 8.0]	1.0 (0.0 to 2.0) [0.0 to 9.0]	NA
Missing	9	6	NA
No. of days prescribed antibiotics for UTI			
Adjusted median difference (95% CI)^a^	−3.00 (−4.40 to −1.60)		<.001
Mean (SD)	5.7 (12.49)	6.3 (10.28)	NA
Median (IQR) [range]	0.0 (0.0 to 7.0) [0.0 to 161.0]	3.0 (0.0 to 7.0) [0.0 to 66.0]	NA
Missing	9	6	NA
Defined daily dose			
Adjusted mean difference (95% CI)^e^	−0.10 (−0.23 to 0.02)		.11
Mean (SD)	0.6 (0.72)	0.7 (0.86)	NA
Median (IQR) [range]	0.0 (0.0 to 1.0) [0.0 to 3.9]	0.0 (0.0 to 1.0) [0.0 to 5.1]	NA
Missing	9	6	NA
Report of consumption of antibiotics			
Adjusted relative risk (95% CI)^d^	0.83 (0.66 to 1.04)		.10
Yes, No. (%)	86 (36.3)	100 (43.9)	NA
No, No. (%)	151 (63.7)	128 (56.1)	NA
Missing	66	67	NA
Count of consumption of antibiotics			
Adjusted incidence rate ratio (95% CI)^d^	0.73 (0.51 to 1.04)		.08
Mean (SD)	2.1 (4.01)	2.9 (5.31)	NA
Median (IQR) [range]	0.0 (0.0 to 3.0) [0.0 to 27.0]	0.0 (0.0 to 4.0) [0.0 to 29.0]	NA
Missing	66	67	NA

Abbreviations: NA, not applicable; UTI, urinary tract infection.

^a^ Quantile regression modeled against randomized arm.

^b^ Mixed-effects Cox proportional hazards model modeled against randomized arm as a fixed effect and site as a random effect.

^c^ Two participants were excluded from the analysis due to having a next consultation with a clinically suspected UTI on the same day as randomization.

^d^ Log-Poisson generalized linear mixed-effects model with robust standard errors modeled against randomized arm as a fixed effect and site as a random effect.

^e^ Linear mixed-effects model modeled against randomized arm as a fixed effect and site as a random effect.

The number of prescribed antibiotic courses for UTI did not differ between the 2 groups. The median number of days of prescribed antibiotics was higher in the placebo group (adjusted median difference, −3.00; 95% CI, −4.40 to −1.60; *P* < .001); however, these outcomes were skewed by a small number of women with high antibiotic consumption during the 6 months of the study (≤161 days in the d-mannose group and 66 days in the placebo group, as detailed in eTable 6 in Supplement 3, along with variation in symptom duration and clinically suspected UTIs). A post hoc sensitivity analysis (eTable 7 in Supplement 3) excluding women with a number of days of prescribed antibiotics more than 3 SDs from the mean (12 women) demonstrated no statistically significant difference between the d-mannose and placebo groups (mean [SD], 4.7 [7.1] days vs 5.1 [7.5] days; adjusted median difference, 0.00; 95% CI, −1.40 to 1.40; *P* > .99; Table 3). Participants in the d-mannose group did not consume fewer antibiotic defined daily doses overall or when split by antibiotic class (Table 3 and eTable 8 in Supplement 3). The proportion of women who reported taking antibiotics at least once in 6 months and the number of times women who reported taking antibiotics during this period were also not significantly different. The proportion of women with resistant uropathogens cultured during an episode of infection was similar (41 of 294 [13.9%] in the d-mannose group and 45 of 289 [15.6%] in the placebo group; RR, 0.90; 95% CI, 0.60-1.33; *P* = .59).

There were similar numbers of hospital admissions related to UTIs in both groups (6 of 294 [2.0%] in the d-mannose group and 4 of 289 [1.4%] in the placebo group; RR, 1.47; 95% CI, 0.47-4.61; *P* = .51). Overall, 28 serious adverse events were reported (20 in the d-mannose group and 8 in the placebo group). None were judged to be related to the intervention. The proportion of participants in the d-mannose group vs placebo group who believed that they had been assigned to d-mannose at 6 months was similar (75 of 224 [33.5%] vs 64 of 215 [29.8%]; odds ratio, 1.19; 95% CI, 0.79-1.78; *P* = .40).

## Discussion

### Summary of Findings

This double-blind, randomized, placebo-controlled trial provides strong evidence that d-mannose did not reduce the proportion of women with a history of rUTI experiencing a further UTI for which they contacted ambulatory care, with 95% CIs excluding the minimum treatment effect considered important by the patient advisory panel. This was unchanged when we compared premenopausal and postmenopausal status and those with higher vs lower numbers of previous UTIs or in sensitivity analyses, including per protocol analyses. The secondary outcomes were similar in both groups: we found no difference in symptom burden, time to the next UTI or number of UTIs, antibiotic use, hospitalizations, or serious adverse events. A similar proportion of women in both study groups believed themselves to have been allocated to d-mannose.

### Comparison With Other Literature

Four systematic reviews of the evidence for d-mannose in treatment of rUTI were published in 2022.^15,22,23,24^ Of these, the Cochrane review concluded that there was currently little to no evidence to support or refute the use of d-mannose to prevent UTIs in all populations.^15^ The authors identified 2 randomized studies examining d-mannose as sole agent prophylaxis for uncomplicated rUTI in women recruited from secondary care settings. The larger study^16^ included 308 women with acute cystitis and a history of rUTI in an open randomized 3-arm trial that compared 2 g of d-mannose, 50 mg of nitrofurantoin, or usual care. Compared with 60% of participants experiencing recurrence rate in the usual care group, 14.6% of participants randomized to d-mannose prophylaxis and 20.4% of participants randomized to nitrofurantoin prophylaxis experienced recurrence. The second study was a randomized crossover trial^25^ of 60 women with rUTI and an acute UTI at the time of recruitment, allocated to either 1 g of d-mannose 3 times daily for 2 weeks followed by 1 g twice daily for 22 weeks, or co-trimoxazole 160 mg/800 mg daily for 1 in every 4 weeks for 24 weeks. The mean time to recurrence was longer in the d-mannose group (200 days vs 52.7 days).

Possible reasons for differences in the present study’s findings include the previous lack of placebo comparison, which could have had an effect on symptomatic presentation rates, and lower incidence of recurrent episodes in a population recruited in primary care. Both studies included women at the point of symptomatic urinary tract infection,^16,25^ while this study included women who had met the criteria for rUTI in terms of previous infections. Research in animal models^26^ demonstrated that rUTI may result from reemergence of intracellular bacterial colonies. d-Mannose initiated during an active infection may have an effect on subsequent infections by reducing or preventing colony formation. Finally, participants in these studies were younger than in the present trial (age range, 29-58 years and 22-54 years vs 18-93 years), and we were not powered to detect differences in subgroups, including those who were premenopausal.

The recurrence rate was higher than that observed in a recent cranberry prophylaxis trial.^21^ This study also used a clinical rather than microbiological primary outcome and recruited from community settings, but clinical confirmation of UTI was mainly investigator led rather than via clinical record review, which could underlie the difference.

### Implications

d-Mannose is a relatively expensive food supplement that is sold directly to the public in the US and UK. It should not be used to prevent rUTI in a primary care setting. However, the present results are not necessarily generalizable to secondary care populations who may be more severely affected. There has recently been interest in other approaches to block the binding of the *Escherichia coli* type 1 pili. Synthetic mannosides, which have a much higher affinity for the FimH adhesin, have shown promise in mouse models.^27,28^ A phase 1 trial of a vaccine against the FimH adhesin recently demonstrated safety and immunogenicity.^29^

### Strengths and Limitations

Strengths of this study include the placebo-controlled design, the sample drawn from a diverse primary care with a population representative of the intended-use population, and 97.5% ascertainment of the primary outcome. Limitations include possible underdosing, although the dose was the same as in 2 previous trials that found benefit.^16,25^ Although the patient advisory panel favored a powder over capsule form, it is possible that capsules might have resulted in a more consistent dose per day. The primary outcome was a pragmatic one of clinically suspected UTI following ambulatory care attendance, chosen with the patient advisory panel as the most important indicator of symptom burden, and we did not seek microbiological confirmation for all recurrence episodes. However, urine cultures were performed at more than 60% of the clinical contacts for suspected UTI, and the proportion of women experiencing at least 1 microbiologically confirmed UTI was similar in both groups. Although more than two-thirds of women reported taking the study product at least 3 days a week for 15 weeks, adherence could have affected the validity of findings. The per protocol analyses including only those women reporting good adherence found similar effects, and adherence was similar in the 2 arms. Finally, a small number of participants experienced outlying durations of symptoms and used antibiotics often, which could have skewed the results. We used post hoc analyses to interrogate the effect of this on findings, which did not meaningfully alter our estimates.

## Conclusions

In this randomized clinical trial, daily d-mannose did not reduce the proportion of women with rUTI in primary care who experienced a subsequent clinically suspected UTI. Daily d-mannose should not be recommended to prevent future episodes of clinically suspected UTI in women with rUTI in primary care.

## Notes

Supplement 1. Trial Protocol

Supplement 2. Statistical Analysis Plan

Supplement 3. **eTable 1.** Additional Baseline Characteristics **eTable 2.** Summary statistics for the sensitivity analyses and the treatment difference between the randomised groups **eTable 3.** Summary statistics for the subgroup analyses and the treatment difference between the randomised groups **eTable 4.** Results from the complier-average causal effect (CACE) analysis **eTable 5.** Details of clinically reported UTIs, urine cultures requested and microbiologically confirmed UTIs by randomized group **eTable 6.** Duration of symptoms and antibiotic use in days, and number of clinically suspected UTIs by study arm **eTable 7.** Post Hoc exploratory analysis comparing the number of days antibiotic use, excluding women with a number of days of antibiotics prescription over 3 standard deviations from the mean **eTable 8.** Mean number of courses and Mean Defined Daily Dosage of antibiotics consumed split by type

Supplement 4. Data Sharing Statement
