# Supplementary material for: d-Mannose for Prevention of Recurrent Urinary Tract Infection Among Women: A Randomized Clinical Trial
Source: JAMA Intern Med. 2024 Apr 8;184(6):619–28. doi: 10.1001/jamainternmed.2024.0264 (PMC11002776; doi:10.1001/jamainternmed.2024.0264)
Supplement: Supplement 1. — Trial Protocol [file jamainternmed-e240264-s001.pdf]

Trial Title: D-mannose to prevent Recurrent UTI: a double blind randomised placebo-controlled trial

Short title: MERIT: d-MannosE to prevent Recurrent urine InfecTions

Ethics Ref: 18/SW/0245

IRAS no.: 245539

Date and Version No: V6.0 17 July 2020

**Chief Investigator:**	Dr Gail Hayward
**Investigators:**	Professor Chris Butler, University of Oxford
	Professor Alastair Hay, University of Bristol
	Professor Michael Moore, University of Southampton
	Dr Yaling Yang, University of Oxford
	Ms Nicola Williams, University of Oxford
	Ms Johanna Cook, University of Oxford
	Ms Julie Allen, University of Oxford
	Dr Margaret Glogowska, University of Oxford
	Dr Nick Thomas, Thames Valley and South Midlands CRN
	Dr Marloes Franssen, University of Oxford
	Mr Jared Robinson, University of Oxford
**Sponsor:**	University of Oxford<br>Joint Research Office<br>1st floor, Boundary Brook House<br>Churchill Drive, Headington<br>Oxford, OX3 7LQ
**Funder:**	NIHR School for Primary Care Research

Chief Investigator Signature:

Statistician Signature:

© Copyright: The University of Oxford and Oxford University Hospitals NHS Foundation Trust 2016

The Investigators declare that there are no conflicts of interest.

Confidentiality Statement

This document contains confidential information that must not be disclosed to anyone other than the Sponsor, the Investigator Team, HRA, host organisation, and members of the Research Ethics Committee, unless authorised to do so.

© Copyright: The University of Oxford and Oxford University Hospitals NHS Foundation Trust 2016

TABLE OF CONTENTS

1. KEY TRIAL CONTACTS 5
2. SYNOPSIS 6
3. ABBREVIATIONS 7
4. BACKGROUND AND RATIONALE 9
5. OBJECTIVES AND OUTCOME MEASURES 10
6. TRIAL DESIGN 12
7. PARTICIPANT IDENTIFICATION 12
   7.1. Trial Participants 12
   7.2. Inclusion Criteria 12
   7.3. Exclusion Criteria 13
8. TRIAL PROCEDURES 13
   8.1. Screening 13
   8.2. Participant identification 13
   8.3. Participant eligibility assessment 14
   8.4. Informed consent 14
   8.5. Qualitative procedures 15
   8.6. Randomisation blinding and code breaking 15
   8.7. Baseline assessments 16
   8.8. Subsequent contact 17
   8.9. Sample handling 19
   8.10. Discontinuation/withdrawal of participants from study treatment 20
   8.11. Definition of End of Study 20
9. Study product (SP) 20
   9.1. SP Description 20
   9.2. Storage of IMP 20
   9.3. Compliance with Trial Treatment 21
   9.4. Accountability of the Trial Treatment 21
   9.5. Concomitant Medication 21
   9.6. Post-trial Treatment 21
10. SAFETY REPORTING 22
   10.1. Definitions 22
   10.2. Causality 23
   10.3. Procedures for Recording Adverse Events 23
   10.4. Reporting Procedures for Serious Adverse Events 23

© Copyright: The University of Oxford and Oxford University Hospitals NHS Foundation Trust 2016

10.5. Expectedness 24
10.6. Safety Monitoring Committee 24
11. STATISTICS 24
11.1. Description of Statistical Methods 24
11.2. The Number of Participants 24
11.3. The Level of Statistical Significance 25
11.4. Criteria for the Termination of the Trial 25
11.5. Procedure for Accounting for Missing, Unused, and Spurious Data. 25
11.6. Inclusion in Analysis 25
11.7. Procedures for Reporting any Deviation(s) from the Original Statistical Plan 26
11.8. Qualitative Interviews and Analysis 26
11.9. Health Economics Analysis 26
12. DATA MANAGEMENT 27
12.1. Source Data 27
12.2. Access to Data 27
12.3. Data Recording and Record Keeping 27
13. QUALITY ASSURANCE PROCEDURES 28
14. SERIOUS BREACHES 28
15. ETHICAL AND REGULATORY CONSIDERATIONS 28
15.1. Declaration of Helsinki 28
15.2. Guidelines for Good Clinical Practice 29
15.3. Approvals 29
15.4. Reporting 29
15.5. Participant Confidentiality 29
15.6. Expenses and Benefits 29
15.7. Other Ethical Considerations 29
16. FINANCE AND INSURANCE 30
16.1. Funding 30
16.2. Insurance 30
17. PUBLICATION POLICY 30
18. DISSEMINATION 30
19. DEVELOPMENT OF A NEW PRODUCT / PROCESS OR THE GENERATION OF INTELLECTUAL PROPERTY 30
20. ARCHIVING 30
21. REFERENCES 31

© Copyright: The University of Oxford and Oxford University Hospitals NHS Foundation Trust 2016

22. APPENDIX A: TRIAL FLOW CHART 33
23. APPENDIX B: SCHEDULE OF PROCEDURES 34
24. APPENDIX C: AMENDMENT HISTORY 36

1. KEY TRIAL CONTACTS

**Chief Investigator**	Dr Gail Hayward,<br>Department of Primary Care Health Sciences<br>Radcliffe Observatory Quarter<br>Woodstock Road<br>Oxford<br>OX2 6GG
**Sponsor**	Ms Heather House,<br>Joint Research Office<br>1st floor, Boundary Brook House<br>Churchill Drive, Headington<br>Oxford<br>OX3 7GB<br>ctrg@admin.ox.ac.uk<br>Tel: +44 (0)1865 572224<br>Fax: +44 (0)1865 572228
**Clinical Trials Unit**	Primary Care Clinical Trials Unit,<br>Department of Primary Care Health Sciences<br>Radcliffe Observatory Quarter<br>Woodstock Road<br>Oxford<br>OX2 6GG<br>merit@phc.ox.ac.uk
**Statistician**	Ms Nicola Williams,<br>Primary Care Clinical Trials Unit<br>Department of Primary Care Health Sciences<br>Radcliffe Observatory Quarter<br>Woodstock Road<br>Oxford<br>OX2 6GG<br>nicola.williams@phc.ox.ac.uk
**Committees**	Trial Steering Committee Chair: Rebecca Cannings-John,<br>Cardiff University<br>412, 4th floor<br>Neuadd Meirionnydd<br>Heath Park<br>Cardiff<br>CF14 4YS<br>canningsrl@cardiff.ac.uk

© Copyright: The University of Oxford and Oxford University Hospitals NHS Foundation Trust 2016

2. SYNOPSIS

Trial Title	MERIT: d-MannosE to prevent Recurrent urine InfecTions	
Internal ref. no. (or short title)	GH/MERIT	
Clinical Phase	Phase IV	
Trial Design	Two arm, individually randomised, double blind placebo controlled trial	
Trial Participants	Women, aged ≥ 18 years old, presenting to ambulatory care with symptoms consistent with urinary tract infections	
Planned Sample Size	Up to 598 Participants	
Treatment duration	24 weeks	
Planned Trial Period	18 months	
	Objectives	Outcome Measures
Primary	To assess the effectiveness of D-mannose in preventing symptomatic UTI	The proportion of women experiencing at least one further episode of clinically suspected UTI for which they contact ambulatory care (e.g. Out of hour (OOH) primary care, in hours primary care, ambulance or accidents and emergency) (assessed by medical notes review) within 26 weeks of study entry.
Secondary	To determine whether daily use of D-mannose compared with placebo by women who experience RUTIs reduces the symptom burden of UTI	-Number of days of moderately bad (or worse) symptoms of UTI (participant diary)<br>-Time to next consultation with a clinically suspected UTI<br>-Number of clinically suspected UTIs<br>-Number of microbiologically proven UTIs
	To determine whether daily use of D-mannose compared with placebo by women who experience RUTIs reduces antibiotic prescription for UTI	-Number of antibiotic courses for UTI; DDD and total mg by antibiotic type<br>-Report of consumption of antibiotics using diary during periods of infection
	To determine whether daily use of D-mannose compared with placebo by women who experience RUTIs can reduce incidence of antibiotic resistant UTIs	Proportion of women with a resistant uropathogen cultured during an episode of acute infection
	To determine the effect of daily use of D-mannose compared with placebo by women who experience RUTIs on hospital admissions related to UTI	Hospital admissions related to UTI

© Copyright: The University of Oxford and Oxford University Hospitals NHS Foundation Trust 2016

	To determine whether daily use of D-mannose compared with placebo by women who experience RUTIs is cost effective	Quality of life and healthcare utilisation data collection
	To determine whether daily use of D-mannose compared with placebo by women who experience RUTIs is considered acceptable and worthwhile by participants	Acceptability and process evaluation conducted via telephone interviews with up to 35 women
Tertiary	To understand the association between previous antibiotic prescribed and presence of bacteria resistant to antibiotics in urinary tract infections in women who experience recurrent UTI	Antibiotic usage and urine culture results in the study period<br>Urine culture results for samples sent during the study period
	To understand in more detail the microbiology of RUTI, including whether women may carry bacteria in their bladders when asymptomatic and the impact of D-mannose. This is an additional exploratory analysis dependent on further funding	Dependent on further funding: Patterns of microbial presence as demonstrated by standard urine culture techniques and next Generation DNA Sequencing in RUTI, exploring association between frequency of infection and microbial presence, relationship between asymptomatic and symptomatic microbial presence in the bladder and on the perineum, and evaluating the impact of D-mannose on microbial presence.
Study Product	D-Mannose	
Formulation, Dose, Route of Administration	Formulation: D-mannose powder (active) or fructose powder (placebo)<br>Dose: 1.5 - 2.5 grams daily<br>Route of administration: Oral	

3. ABBREVIATIONS

A&E	Accidents and emergency
AE	Adverse event
AR	Adverse reaction
cfu/ml	Colony forming unit /millilitre
CI	Chief Investigator
CRF	Case Report Form
CRN	Clinical Research Network

© Copyright: The University of Oxford and Oxford University Hospitals NHS Foundation Trust 2016

CTRG	Clinical Trial and Research Governance
CUA	Cost-utility analysis
DDD	Defined Daily Dose
DMP	Data Management Plan
DPA	Data Protection Act
EU	European Union
GDPR	General Data Protection Regulation
GCP	Good Clinical Practice
GP	General Practitioner
HRA	Health Research Authority
ICF	Informed Consent Form
ICH	International Conference on Harmonisation
IMP	Investigational Medicinal Product
MHRA	Medicines and Healthcare products Regulatory Agency
NHS	National Health Service
NIHR	National Institute for Health Research
OOH	Out of Hours
OTC	Over the Counter
PC-CTU	Primary Care Clinical Trials Unit
PIS	Participant/Patient Information Sheet
R&D	NHS Trust R&D Department
REC	Research Ethics Committee
RUTI	Recurrent Urinary Tract Infection
SAE	Serious Adverse Event
SAR	Serious Adverse Reaction
SOP	Standard Operating Procedure
SP	Study Product

© Copyright: The University of Oxford and Oxford University Hospitals NHS Foundation Trust 2016

SUSAR	Suspected Unexpected Serious Adverse Reactions
QALYs	Quality adjusted life years
UK	United Kingdom
UTI	Urinary Tract Infection

4. BACKGROUND AND RATIONALE

Urinary tract infections (UTIs) are the commonest bacterial infection seen in women presenting to UK primary care (1, 2), accounting for up to 3% of all GP consultations (3). Approximately 40-50% of women experience one episode during their lives. (3) Recurrent UTIs (RUTIs) are commonly defined in the literature as three episodes of UTIs in the last twelve months or two episodes in the last six months (4). Between 20-44% of women who have had one episode of UTI will have a further UTI and around 25% of these will develop subsequent recurrent episodes (5-7). In a recent survey of 2424 randomly selected UK women, 3% reported RUTI in the past year, equivalent to 800000 women annually in the UK (8). RUTIs have a significant negative impact on quality of life which extends beyond the unpleasant symptomatology into distressing and disrupted sexual relationships, persistent unmanageable pain and systemic illness (9). They have a high impact on health care costs as a result of outpatient visits, diagnostic tests and prescriptions (8). A recent Italian study estimated direct costs per episode of RUTI at 142 euros (approximately £125) (10).

To date, antibiotic prophylaxis is the only treatment which has been demonstrated to be beneficial for RUTIs in a systematic review of randomised controlled trials, although a recent large study suggests some benefit from cranberry juice (11). Antibiotics given continuously for six to twelve months were better than placebo at preventing recurrent infection (NNT 1.85) (4), and national guidelines advocate their use (12). However, antibiotics also resulted in more severe and unpleasant side effects (e.g. vomiting, urticaria, candidiasis). Furthermore, once prophylaxis is discontinued, even after extended periods, approximately 50-60% of women will become re-infected within three months (13, 14). Thus, antibiotic prophylaxis does not exert a long-term effect on the baseline infection rate and antibiotic use is directly linked to antibiotic resistance in uropathogens (15). Antibiotic resistance has been positively associated with an increased duration of severe symptoms of UTIs, irrespective of the use of an appropriate antibiotic (2, 15).

D-mannose is a type of sugar (a monosaccharide isomer of glucose) which is considered to inhibit bacterial adherence to uroepithelial cells by binding to a site on the tip of the fimbria (16) and has shown benefit in animal models (17). It is an essential glyconutrient for human health. It is an important metabolic intermediate product in the biosynthesis of most secretory proteins and glycoproteins in the human body (18). It is absorbed in the upper gastrointestinal tract and excreted in the urine, and the proposed daily dose is equivalent to less than 2 grams of glucose (19), comparing favourably to cranberry juice, which contains 22 grams of sugar per 200ml. It can be found naturally within the diet occurring in numerous food sources, such as coffee, baker's yeast, egg white, fruits such as apples, cranberries, and mangos and also in legumes such as soybeans, kidney beans and peanuts (20).

Currently D-mannose is available as a food supplement which is favoured by women who have RUTIs, but until recently has had little empirical evidence to support its use. An open label randomised three arm trial including 308 women with RUTI seen in outpatient settings (19), showed that daily use of D-mannose for

© Copyright: The University of Oxford and Oxford University Hospitals NHS Foundation Trust 2016

six months resulted in an absolute reduction in incidence of further urinary tract infection by 45% from a rate of 60% in the usual care arm, with no adverse events. Incidence was reduced by 11% compared to daily antibiotic use. This finding is supported by two recent smaller studies. A feasibility study in 20 women with Multiple sclerosis found a reduction in monthly UTI rate with D-mannose use. Over 80% of participants wished to continue using the product beyond the study period (20). A small cross-over pilot study in 60 women presenting to urology outpatient clinics found a longer duration to relapse (52 v 200 days) in those taking D-mannose (21).

Although there are early indicators of efficacy, the only large study to date (19) was not placebo controlled and had very high UTI incidence in the control arm. Furthermore, a microbiologically confirmed UTI was a requirement for entry to the study, and participants were withdrawn once they developed a UTI on treatment, meaning true incidence of UTI could not be established, a very important measure for women who experience very frequent UTIs, who are also the most likely candidates for prophylaxis. Finally, all women on hormonal contraception were excluded, which reduces applicability to the UK female population at highest risk.

D-mannose has the potential to offer a valuable alternative to antibiotic prophylaxis in women who experience RUTI and in turn to contribute to better antimicrobial stewardship in primary care. It has been suggested to us as a key target for a clinical trial by the patients who suffer with this condition, some of whom find it highly beneficial and all of whom would welcome a high-quality addition to evidence base for this product. The high costs (at least £25 a month) associated with its purchase add weight to the need to establish whether this is treatment which GPs should be advising their patients to pursue.

In order to ensure that women taking part in the trial are receiving a sufficient amount of D-mannose for its proper investigation without requiring alteration to their normal diet we will provide a standardised amount of D-mannose powder; however due to the varying density of the study product this may vary between 1.5 and 2.5 grams. This study will evaluate the efficacy of D-mannose in women suffering with RUTI presenting to UK primary care and its cost effectiveness.

5. OBJECTIVES AND OUTCOME MEASURES

**Objectives**	**Outcome Measures**	**Time point(s) of evaluation of this outcome measure (if applicable)**
**Primary Objective**<br>To assess the effectiveness of daily use of D-mannose compared with placebo in preventing symptomatic UTI in women	The proportion of women experiencing at least one further episode of clinically suspected UTI for which they contact ambulatory care (Out of hour (OOH) primary care, in hours primary care, ambulance or A&E) within 26 weeks of study entry.	26 weeks after study entry (notes review)
**Secondary Objectives**<br>To determine whether daily use of D-mannose compared with placebo	Number of days of moderately bad (or worse) symptoms of UTI (participant diary)	Throughout study (participant diary)

© Copyright: The University of Oxford and Oxford University Hospitals NHS Foundation Trust 2016

by women who experience RUTIs reduces the symptom burden of UTI	Time to next consultation with a clinically suspected UTI<br><br>Number of clinically suspected UTIs<br><br>Number of microbiologically proven UTIs	26 weeks after study entry (notes review)
To determine whether daily use of D-mannose compared with placebo by women who experience RUTIs reduces antibiotic prescription for UTI	Number of antibiotic courses for UTI; Defined daily dose (DDDs) and total mg by antibiotic type<br><br>Report of consumption of antibiotics using diary during periods of infection	Number of antibiotic courses: 26 weeks after study entry (notes review)<br><br>Consumption of antibiotics: Throughout study (participant diary)
To determine whether daily use of D-mannose compared with placebo by women who experience RUTIs can reduce incidence of antibiotic resistant UTIs	Proportion of women with a resistant uropathogen culture during an episode of acute infection	26 weeks after study entry (notes reviews)
To determine the effect of daily use of D-mannose compared with placebo by women who experience RUTIs on hospital admissions related to UTI	Hospital admissions related to UTI	26 weeks after study entry (notes review)
To determine whether daily use of D-mannose compared with placebo by women who experience RUTIs is cost effective	Quality of life and healthcare utilisation data collection	Quality of life: baseline, throughout study and during UTI episodes on day 1, 3 and 5 (participant diary).<br><br>Healthcare utilisation: 26 weeks after study entry (notes review)
To determine whether daily use of D-mannose compared with placebo by women who experience RUTIs is considered acceptable and worthwhile by participants	Acceptability and process evaluation conducted via telephone interviews with up to 35 women	Throughout the study (optional qualitative interviews)

© Copyright: The University of Oxford and Oxford University Hospitals NHS Foundation Trust 2016

**Tertiary Objectives**<br>To understand the association between previous antibiotic prescribed and presence of bacteria resistant to antibiotics in urinary tract infections in women who experience recurrent UTI	Antibiotic usage in the study period<br><br>Urine culture results for samples sent during the study period	Antibiotic usage: Notes review<br>Urine culture results: Throughout the study
To understand in more detail the microbiology of RUTI, including whether women may carry bacteria in their bladders when asymptomatic and the impact of D-mannose. This is an additional exploratory analysis dependent on further funding	Dependent on further funding: Patterns of microbial presence as demonstrated by standard urine culture techniques and next Generation DNA Sequencing in RUTI, exploring association between frequency of infection and microbial presence in the bladder and on the perineum, relationship between asymptomatic and symptomatic microbial presence, and evaluating the impact of D-mannose on microbial presence.	Throughout study

6. TRIAL DESIGN

This will be a two arm, individually randomised, double blind placebo controlled, pragmatic trial, evaluating a complex strategy which involves advice to take a food supplement according to the manufacturers advice. The trial will approximate the intervention as closely as possible to what would occur in the real world. Participants will be randomised 1:1 and will be enrolled for 24 weeks. After completion, notes reviews will be completed by a GP or nurse to collect primary and secondary outcome data. A subgroup of women from both trial arms will be interviewed after they have completed the main trial and finished taking the study product.

See Appendix A for Study Flowchart.

7. PARTICIPANT IDENTIFICATION

7.1. Trial Participants

Female participants presenting to ambulatory care with RUTIs

7.2. Inclusion Criteria

- Participant is willing and able to give informed consent for participation in the study
- Participant is able to comply with study procedures
- Female, aged 18 years or above
- Presented to ambulatory care with symptoms consistent with UTI (search will be based on UTI / suspected UTI / UTI specific antibiotics) three or more times in the last year or two or more times in the last six months

© Copyright: The University of Oxford and Oxford University Hospitals NHS Foundation Trust 2016

7.3. Exclusion Criteria

The participant may not enter the trial if ANY of the following apply:

- Female participant who is pregnant, lactating or planning pregnancy during the course of the study
- Formal diagnosis of interstitial cystitis or overactive bladder syndrome
- Prophylactic antibiotics started in the last three months and unwilling to discontinue, or intention to start during the next six months
- Currently using D-mannose and unwilling to discontinue for the duration of the study
- Nursing home resident (residential home residents will not be excluded)
- Catheterised, including intermittent self-catheterisation
- Use of Uromune (an 'immunostimulant')
- Participation in a research study involving an investigational medicinal product (IMP) in the past twelve weeks
- Previous participation in this study

8. TRIAL PROCEDURES

8.1. Screening

Screening will involve the direct care team using a medical records search, repeated periodically, to identify potentially eligible participants. The GP will then determine eligibility against the inclusion and exclusion criteria as outlined above to refine a list of potential participants to be contacted. If the participant has responded to the advert (see below), the GP will receive a letter after baseline to confirm participant's eligibility.

8.2. Participant identification

Appendix B provides an outline of the trial procedures and time points.

Up to 35 participants will be contacted to take part in qualitative interviews as detailed below. Consent for further contact regarding the interviews will be taken at the baseline appointment. This will be optional.

8.2.1 Postal or Telephone invitations

The primary method of identification will be electronic primary care medical record screening followed by telephone and / or postal invitation by the GP surgery or study team (this includes members of the clinical research network (CRN)). If participants confirm their interest in taking part in the study during the telephone call, they will then they will be sent a participant information sheet (PIS) by post or email.

8.2.2 Opportunistic recruitment

Participants will also be identified when they present themselves to their GP Surgery with symptoms of a UTI in which case they will be given a PIS. Advertisements will be used in GP surgeries, minor injury units and other community healthcare settings as a secondary method of identification. These adverts will either be placed in a GP surgery or, if placed in other community healthcare settings will list participating local surgeries which the participant will need to be registered with in order to take part. Potential participants

© Copyright: The University of Oxford and Oxford University Hospitals NHS Foundation Trust 2016

responding to the adverts will be contacted by the study team and eligibility will be checked with their GP after they have consented to this.

8.3. Participant eligibility assessment

The participant can return their expression of interest via freepost to the study team; can email or call the study team directly; or can go online to complete their expression of interest via a link provided to them in the PIS. After receiving the expression of interest the team will send the participant an initial email confirming receipt of the expression of interest and to book in a time for the face to face or telephone appointment. The participant will also be sent a link where they can complete an eligibility assessment, informed consent form and baseline questionnaire online ahead of the face to face or telephone appointment, however, they do not have to complete this prior to the appointment. They will be given an access code to these forms that needs to be used specific with their name in order to be able to enter the online forms.

8.3.1 Face to face

If the participant is being recruited via a face to face appointment at their GP surgery the suitably qualified clinician (GP or research nurse) will confirm eligibility at the point of recruitment into the study and obtain informed consent from the participant in accordance with 8.4.1.

8.3.2 Phone

During the baseline appointment over the phone with the research team, participants will confirm their eligibility. Potential participants responding to the adverts will be contacted by the study team and eligibility will be checked with their GP after they have consented to this.

8.3.3 Online

In the PIS the participants will be provided with a link to a website where they can complete an eligibility assessment online. Potential participants responding to the adverts will be contacted by the study team and eligibility will be checked with their GP after they have consented to this.

8.4. Informed consent

The participant must personally sign and date (either in ink or using electronic methods) the latest approved version of the Informed Consent Form (ICF) before any study specific procedures are performed.

8.4.1 Face to face consent

Written and verbal versions of the PIS and ICF will be presented to the participants detailing no less than: the exact nature of the trial; what it will involve for the participant; the implications and constraints of the protocol; the known side effects and any risks involved in taking part. It will be clearly stated that the participant is free to withdraw from the study at any time, for any reason, without prejudice to future care, without affecting their legal rights and with no obligation to give the reason for withdrawal. The participant will be allowed as much time as they require to consider the information, and the opportunity to question the investigator, their GP or other independent parties to decide whether they will participate in the trial. Written or electronic Informed Consent will then be obtained by means of participant dated signature and dated signature of the person who presented and obtained the Informed Consent. The person who

© Copyright: The University of Oxford and Oxford University Hospitals NHS Foundation Trust 2016

obtained the consent must be suitably qualified and experienced, and have been authorised to do so by the Chief/Principal Investigator. Once ICF is signed a copy will be given to the participant and a copy will be sent via secure email to the participants' GP and stored with the participants' notes.

8.4.2 Consent documented during or after the phone call

Participants who have completed eligibility over the phone will be given a link to the online ICF. They can complete the ICF electronically at the time of the telephone call once they are happy to do so. Participants will be able to download a copy of the consent form to keep for their records; the research team will store a copy of the consent form and will send the practices a copy (secure via NHS email or post) for storage in the participants' notes.

8.4.3 Consent documented online

After completing the online eligibility assessment, participants will be referred to the online consent form. Participants will be able to download a copy of the consent form to keep for their records; the research team will store a copy of the consent form and will send the practices a copy (via secure NHS email or post) for storage in the participants' notes.

8.5. Qualitative procedures

During the consent process for the main trial participants will be asked if they are happy to be contacted about taking part in the qualitative work. A sub-group of those who do consent will be contacted separately by a qualitative researcher, and offered further information about the qualitative interviews using a separate PIS. This researcher will have access to their allocation after participants have finished taking the study product, in order to select an appropriate sample of participants. When they have had chance to consider it and if they are still willing to participate, a convenient time for the interview, that will last approximately 30 minutes, will be arranged. Consent will be verbal and audio-taped, using standard wording. Participants from both study arms will be interviewed after their participation in the trial has finished.

8.6. Randomisation blinding and code breaking

Randomisation will be performed using Sortition, Primary Care Clinical Trial Unit's (PC-CTU) in-house online randomisation system, according to the current version of the standard operating procedure (SOP) PC-CTU_SOP_IT104. Block randomisation will be implemented with varying block sizes. Randomisation will be stratified by practice ensuring a balance of the two arms within each practice. Participants will be randomised to receive 24 weeks of D-mannose or matched placebo using an allocation ratio of 1:1. Neither the participants nor the recruiter will know to which arm they have been randomised. For patients being recruited acutely concealment will be maintained to prevent any treatment decision regarding the UTI following knowledge of the treatment allocation.

If the randomisation system is unavailable an emergency randomisation list will be held at the PC-CTU and this will be used to randomise the participant. This will be done according to the current version of PC-CTU SOP ST05 "Randomisation and Blinding Procedures".

Out of hours' code-breaking will not be required due to the risk level of D-mannose, which is a sugar naturally occurring within the diet. Based on previous study literature and manufacturer literature we do

© Copyright: The University of Oxford and Oxford University Hospitals NHS Foundation Trust 2016

not anticipate any adverse events which would require code breaking. Code breaking will be possible, however, during office hours. The study statistician will inform the necessary clinical team of the study allocation. As far as possible the rest of the study team will remain blind to the allocation. If the clinical condition of a participant necessitates breaking the code, this will be undertaken using the Sortition unblinding process.

The only unblinded members of the team will be the study statistician at final analysis and the qualitative researcher once the participant has finished taking the study product (see 8.5). The study statistician will remain blinded to treatment allocation at interim analysis and unblinded at final analysis.

8.7. Baseline assessments

The baseline assessment will include collection of the following:

- Month and year of birth
- Medical details including: diagnosis and treatment for urinary incontinence, whether they are pre or post-menopausal, hormonal contraception use and hormone replacement therapy
- Detailed history of episodes of UTI over the previous year, including those which were not reported to ambulatory care and symptom burden of each episode (i.e. severity of symptoms during episodes) up to a maximum of 3 episodes
- Known / suspected risk factors for recurrent UTI e.g. sexual activity
- Current usage of preventative medications for UTI
- EQ-5D-5L

8.7.1 Face to face

If the baseline appointment takes place in person at the GP surgery, a GP or research nurse will check eligibility, take consent and complete the baseline case report form (CRF) online. The research team will receive a notification at the end of baseline assessments and will then randomise the participant and send the study product.

8.7.2 Baseline completed during phone call

Following an eligibility check and informed consent, the recruiter will complete the baseline CRF with the participant over the phone. After the phone call, the participant will be randomised and sent the study product.

8.7.3 Online

If the participant decides to complete the baseline online, they will receive a link after they have completed the ICF that takes them to the baseline CRF for them to complete. After completion the study team will contact them to ensure the participant is happy with the study procedures after which the participant will be randomised and sent the study product.

© Copyright: The University of Oxford and Oxford University Hospitals NHS Foundation Trust 2016

8.7.4 Baseline samples

The participant will be asked to send a urine sample when they begin the study (and do not have an active UTI) to the University of Oxford laboratory using the pot and packaging supplied at randomisation. If the participant is experiencing an active UTI when they begin the study, they should wait until two days after symptoms have resolved before collecting a sample.

Participants will also be asked to consider sending a perineal self-swab when they begin the study. This will be optional, and explained to participants in an 'Instructions for participants' leaflet, with the swab kit and packaging supplied at randomisation.

8.8. Subsequent contact

There is no requirement for participants to have face to face follow up appointment as part of their study participation. The follow up period for the study is (26 weeks) from the point of randomisation. During this time each participant will receive postage of D-mannose or placebo every other month, a weekly text message/email, and a monthly phone call if needed. If the participant experiences symptoms they believe to be due to a UTI (including at least one of dysuria, frequency, suprapubic or flank pain or haematuria) during the follow up period, they will be asked to complete a daily symptom diary (online or paper; if completed on paper, a member of team will call the participants to collect the information completed, or a prepaid envelope with be provided if required). Some participants may report constant low level symptoms similar to this with flares. In this case we will only ask them to complete the diary for the times at which symptoms are worse than usual. Participants will be asked to send urine samples to the study laboratory when they develop symptoms of UTI, and two days after these symptoms have resolved.

8.8.1 Weekly text messages/emails

The weekly text message/email will remind people about the use of the online diary should any symptoms develop and will include a link to an online questionnaire collecting the following information:

- Assessment of compliance with study product
- Whether the participant has had any symptomatic UTI episodes, or felt their symptoms are worse than normal, in the case that they experience continuous low level symptoms. If they have, but have not completed the UTI daily symptom diary we will ask them about the number of episodes experienced, level of symptom severity, duration of symptoms, whether they made contact with any health care provider as a result of the episode, whether they took any prescribed or over the counter medication or any other products for the episode.

8.8.2 Monthly phone calls

Monthly phone calls conducted by the research team will be done if weekly questionnaires are not completed and will collect the following:

- An assessment of compliance with study medication
- A reminder regarding the use of the online diary should any symptoms develop.
- Whether the participant has had any symptomatic UTI episodes. If they have, but have not completed the UTI daily symptom diary we will ask them about the:
    - Number of episodes experienced

© Copyright: The University of Oxford and Oxford University Hospitals NHS Foundation Trust 2016

- Level of symptom severity
- Duration of symptoms
- Whether they made contact with any health care provider as a result of the episode
- Whether they took any prescribed or over the counter medication or any other products for the episode

8.8.3 During a UTI

The daily symptom diary completed when the participant experiences a UTI will collect the following information:

Rating of the following symptoms each day in terms of severity:

- Fever
- Burning or pain when passing urine
- Urgency
- Day time frequency
- Night time frequency
- Ability to perform usual activities
- Feeling generally unwell
- Nausea
- Loin pain

Use of over the counter (OTC) medications or 'rescue' antibiotics they had kept back from another episode, but had not been prescribed during the study period

Consumption of antibiotics prescribed by GP
EQ-5D-5L on day 1, 3 and 5
Whether they made contact with any health care provider due to their symptoms of UTI

Participants will also be requested to provide the study team with a urine sample if they develop UTI symptoms and another sample two days after symptoms have resolved. The sample pots and envelopes to send the sample will be sent to the participants with the first mail out of study product. Once the lab confirms receipt of a participant's sample, two additional sample pots and envelopes will be sent to the participant in case they develop another UTI.

8.8.4 Final Questionnaire CRF

After the participant has finished taking the study product, they will be asked to complete a final CRF online, on paper, or over the phone. The CRF will collect the following information:

- Adherence to the study product in the last month
- EQ-5D-5L
- Whether the participant thought they were taking D-mannose or the placebo. This is being asked as part of the qualitative analysis

© Copyright: The University of Oxford and Oxford University Hospitals NHS Foundation Trust 2016

8.8.5 Notes reviews

Notes review will be conducted for all participants by the GP or (research) nurse (this includes CRN research nurses), unless a participant explicitly withdraws their consent for this following withdrawal from the trial, and should take around 30 minutes to complete. This will record:

- Presentations to ambulatory care with symptoms consistent with UTI during the 26 weeks of study participation (including participants who withdraw early or are deemed lost to follow-up)
- Results of urine cultures during the study period
- Medications prescribed during these episodes
- Hospital admissions

8.9. Sample handling

At baseline, when participants are asymptomatic (or have the same mild symptoms as usual), they will be asked to send a urine sample and an optional perineal swab to the University of Oxford laboratory using the packaging supplied at or after their randomisation. If the participant is recruited when they have an acute UTI they will be asked to wait until at least two days after symptoms have resolved before sending this sample. If the participant develops UTI symptoms during the 24 weeks following recruitment, they will also be requested to send an additional urine sample to our study laboratory at that time and two days after resolution of symptoms. These samples will be

- Cultured using standard NHS laboratory procedures, with bacterial presence recorded to 103 cfu/ml (urine and perineal swab)
- Examined with microscopy to identify epithelial cells, red cells and white cells (urine only)
- Samples will be frozen and stored and if further funding is successfully obtained: Analysed using microbiological techniques including visual qualitative techniques, using microbiological assays to evaluate adhesion and invasion of the urothelium, phylotyping techniques and next generation DNA sequencing techniques. Bacteria identified in perineal and urine samples from women when asymptomatic will be compared with urine culture results from an episode of infection to assess whether the infection could be arising from bacteria carried within the bladder or on the perineum.

Although urine samples are generally assumed to be sterile, there is a small possibility that trace amounts (<0.1%) of human DNA may be found in these samples using sequencing techniques. When the DNA samples are sequenced and electronic DNA sequences generated, we will immediately use an automated computer programme that looks for human DNA and removes it, without performing any analysis, so that stored electronic DNA sequences on University servers, which are subsequently analysed, have these trace amounts removed.

Additional optional consent will be sought for further analyses on de-identified samples including work by commercial parties within the EU and outside.

The samples will be labelled according to laboratory procedures, including participant ID, and date of collection.

© Copyright: The University of Oxford and Oxford University Hospitals NHS Foundation Trust 2016

8.10. Discontinuation/withdrawal of participants from study treatment

Each participant has the right to withdraw from the study at any time. In addition, the investigator may discontinue a participant from the study at any time if the investigator considers it necessary, for any reason including:

- Ineligibility (either arising during the study or retrospectively having been overlooked / unknown at screening)
- Major illness which limits the participants' ability to continue in the study
- Loss to follow up

The reason for withdrawal will be recorded in the CRF.

If a participant withdraws themselves, data already collected will be used in the intention to treat analysis, and a notes review will be performed except when participants explicitly remove consent for this.

If a participant is found to be ineligible after randomisation, they will be removed from the study, however their data will not be removed from the intention to treat analysis. Additional participants will be recruited if required as described in section 11.2.

8.11. Definition of End of Study

The end of the study will be the date that the database has been cleaned and closed.

9. Study product (SP)

9.1. SP Description

Participants will be requested to take a daily dose of either of D-mannose in powder form or of fructose in powder form. This will be dispensed using a 2-gram scoop, but there is the potential that the dose will range between 1.5 and 2.5 grams due to the density of the product.

The products will be provided by Tiofarma BV, a manufacturing chemist in the Netherlands. The SP packets will be labelled according to the manufacturers regulations. Each study product pack will be printed with a unique medication ID number to ensure D-mannose and fructose are indistinguishable and thus maintain allocation concealment.

The study product will be a powder form in 124g pots and will be sent out to participants every other month. A scoop will be provided that measures 2g of the study product.

A Participant Medication Letter may be issued to participants upon request, if they intend to travel abroad and continue to take their study product during this time, in case of inspection of their study product in airport security/customs, to explain the contents of the study product and its purpose.

9.2. Storage of IMP

The study product and the placebo are stored at room temperature. It will be stored by participants at home and by the study team when obtained from the manufacturer.

© Copyright: The University of Oxford and Oxford University Hospitals NHS Foundation Trust 2016

9.3. Compliance with Trial Treatment

Participants will be sent a text message/email weekly to assess their compliance. This will also be confirmed in the monthly CRF if needed. All randomized trial participants will be included in the intention-to-treat population.

9.4. Accountability of the Trial Treatment

SP will be sent to each participant every other month and fully tracked by the study team. Therefore, if a participant withdraws or is lost to follow up no unnecessary SP will be lost. The participants will not be required to return any empty or unused SP. If the participant requires more study product for any reason this will be supplied.

9.5. Concomitant Medication

D-mannose is a food supplement and as such there are no contraindicated medications.

9.6. Post-trial Treatment

There will not be provision of D-mannose beyond the trial period.

© Copyright: The University of Oxford and Oxford University Hospitals NHS Foundation Trust 2016

10. SAFETY REPORTING

10.1. Definitions

Adverse Event (AE)	Any untoward medical occurrence in a participant to whom a medicinal product has been administered, including occurrences which are not necessarily caused by or related to that product.
Adverse Reaction (AR)	An untoward and unintended response in a participant to an investigational medicinal product which is related to any dose administered to that participant.<br>The phrase "response to an investigational medicinal product" means that a causal relationship between a trial medication and an AE is at least a reasonable possibility, i.e. the relationship cannot be ruled out.<br>All cases judged by either the reporting medically qualified professional or the Sponsor as having a reasonable suspected causal relationship to the trial medication qualify as adverse reactions.
Serious Adverse Event (SAE)	A serious adverse event is any untoward medical occurrence that:<br>• results in death<br>• is life-threatening<br>• requires inpatient hospitalisation or prolongation of existing hospitalisation<br>• results in persistent or significant disability/incapacity<br>• consists of a congenital anomaly or birth defect.<br>Other 'important medical events' may also be considered serious if they jeopardise the participant or require an intervention to prevent one of the above consequences.<br>NOTE: The term "life-threatening" in the definition of "serious" refers to an event in which the participant was at risk of death at the time of the event; it does not refer to an event which hypothetically might have caused death if it were more severe.<br>In this study, we will not be classifying planned hospital admissions e.g. elective procedures, as SAEs.
Serious Adverse Reaction (SAR)	An adverse event that is both serious and, in the opinion of the reporting Investigator, believed with reasonable probability to be due to one of the trial treatments, based on the information provided.
Suspected Unexpected Serious Adverse Reaction (SUSAR)	A serious adverse reaction, the nature and severity of which is not consistent with the information about the medicinal product in question set out:<br>• in the case of a product with a marketing authorisation, in the summary of product characteristics (SmPC) for that product

© Copyright: The University of Oxford and Oxford University Hospitals NHS Foundation Trust 2016

	• in the case of any other investigational medicinal product, in the investigator's brochure (IB) relating to the trial in question.

NB: to avoid confusion or misunderstanding of the difference between the terms "serious" and "severe", the following note of clarification is provided: "Severe" is often used to describe intensity of a specific event, which may be of relatively minor medical significance. "Seriousness" is the regulatory definition supplied above.

Any pregnancy occurring during the clinical trial and the outcome of the pregnancy should be recorded and followed up for congenital abnormality or birth defect, at which point it would fall within the definition of "serious".

As D-mannose is an isomer of glucose, pregnancy will not be considered a serious adverse event and will not be followed up until birth. Any woman becoming pregnant during the course of the study will be withdrawn.

10.2. Causality

The relationship of each adverse event to the study product must be determined by a medically qualified individual according to the following definitions:

Unrelated – where an event is not considered to be related to the SP

Possibly – although a relationship to the SP cannot be completely ruled out, the nature of the event, the underlying disease, concomitant medication or temporal relationship make other explanations possible.

Probably – the temporal relationship and absence of a more likely explanation suggest the event could be related to the SP.

Definitely – the known effects of the SP, its therapeutic class or based on challenge testing suggest that the SP is the most likely cause.

All AEs (SAEs) labelled possibly, probably or definitely will be considered as related to the SP.

10.3. Procedures for Recording Adverse Events

As D-mannose is a food supplement and found naturally within the diet, we will not collect any non-serious adverse events.

10.4. Reporting Procedures for Serious Adverse Events

SAEs will be recorded from the first day of use of the intervention to the last day of use.

All SAEs must be reported on the SAE Report Form by the person who has discovered the SAE or nominated delegate. If this person is at a recruiting site they will fax, or scan and email it to the sponsor or delegate (in the UK the PC-CTU) within 24 hours of the site study team becoming aware of the event. The sponsor or delegate will perform an initial check of the report, request any additional information, and ensure it is reviewed by the CI or other delegated personnel for relatedness as soon as possible and log the event on

© Copyright: The University of Oxford and Oxford University Hospitals NHS Foundation Trust 2016

our SAE log. Additional and further requested information (follow-up or corrections to the original case) will be detailed on a new SAE Report Form and faxed/emailed to the sponsor or delegate. If the event has not resolved, at the 24-week time point the SAE will be reviewed again to see if resolution has occurred. If the event is considered 'resolved' or 'resolving' no further follow up is required. If not the event must be followed up until such a time point. All SAEs will be reported annually to the relevant REC.

Planned hospital admissions including elective procedures will not be reported as SAEs.

10.5. Expectedness

We do not anticipate any serious adverse events due to the intervention or the qualitative studies.

10.6. Safety Monitoring Committee

The Trial Steering Committee will additionally act as the safety monitoring committee because of the low risk nature of the study. They will review the SAE log:

- To pick up any trends, such as increases in un/expected events, and take appropriate action
- To seek additional advice or information from investigators where required
- To evaluate the risk of the study continuing and take appropriate action where necessary
- To monitor the overall rate of women developing UTIs

11. STATISTICS

11.1. Description of Statistical Methods

The primary outcome, the proportion of women experiencing at least one further episode of UTI symptoms for which they visited their GP within 24 weeks of study entry, and other binary outcomes, will be analysed on an intention-to-treat basis by means of a generalised linear mixed effects model with binomial distribution and log link function, including a random effect for practice and fixed effect for randomisation group. Therefore, treatment groups will be compared on the basis of an adjusted risk ratio. The number of days of moderately bad symptoms of UTI, the number of UTI's experienced in 24 weeks, and number of antibiotic courses for UTI in 24 weeks, will be analysed by means of a generalised linear mixed effects model using the Poisson distribution and log link function, including a random effect for practice and a fixed effect for randomised group. Defined Daily Doses (DDDs) will be analysed by means of a linear mixed effects model including a random effect for practice and fixed effects for randomised group and baseline DDD, treating this outcome as continuous. We will analyse the overall DDD as well as the individual antibiotic DDDs.

11.2. The Number of Participants

The most recent study to evaluate prophylactic treatment for RUTI in a similar population (Maki et al 2016) found that 26.6% of women in the control arm experienced a UTI within six months. Discussion with our PPI advisors suggests that in order to commit to daily use of a prophylactic regime they would require evidence of at least a 50% reduction in the chance of a further UTI during the period of prophylaxis. To detect this with 90% power and an alpha of 0.05 we would require 203 participants in each arm. This equates to 508 participants if a 20% loss to follow up is assumed. This sample size is also adequate to

© Copyright: The University of Oxford and Oxford University Hospitals NHS Foundation Trust 2016

power the key secondary outcome (the number of UTI's experienced over 24 weeks), and detect a relative incidence rate of 0.5 between the treatment and placebo groups, assuming a base rate of 0.36 as estimated by Maki et al (2016). If the estimated percentage of participants who have either withdrawn or failed to respond to any study team communication for an extended period seems likely to rise above the 20% initially allowed for, we will recruit additional participants, up to a maximum of 598 participants.

11.3. The Level of Statistical Significance

The 5% significance level was used to calculate number of participants required for the study. Significance testing will be performed at the 5% level (2-sided hypothesis tests).

11.4. Criteria for the Termination of the Trial

No futility analysis or interim analysis is planned for this study.

11.5. Procedure for Accounting for Missing, Unused, and Spurious Data.

The missing at random assumption will be tested as far as is possible by analysing each baseline covariate in a regression model to determine which if any are associated with missingness. All baseline covariates are expected to be observed. Baseline values will be summarised for those who did and did not complete follow up measurements to describe any characteristics related to missingness that are able to be observed.

We will be analysing our data using an intention to treat analysis. All randomised participants will be included in the analysis, assuming non-informative censoring for those withdrawn from the study or lost to follow-up for the primary analysis.

During statistical data review and analysis, any anomalies in the data will be investigated and discussed with the study management team. The data investigation will be broad and flexible and focus on variability of the data, consistency, dispersion, outliers, inliers, relationships between variables and relationships over time. The statistical data review will be fully documented with all the output dated. If fraud is proved, fraudulent data will be removed from the analysis.

We will include a sensitivity analysis of the primary outcome which will impute missing data by means of multiple imputation making use of additional covariates collected at baseline, such as age and reported sexual activity.

11.6. Inclusion in Analysis

We will be analysing our data using an intention to treat analysis. All randomised participants will be included in the analysis, assuming non-informative censoring for those withdrawn from the study or lost to follow-up for the primary analysis.

A secondary analysis will be based on a per protocol population to allow for an assessment of those participants who have taken the allocated intervention.

© Copyright: The University of Oxford and Oxford University Hospitals NHS Foundation Trust 2016

11.7. Procedures for Reporting any Deviation(s) from the Original Statistical Plan

We do not anticipate any deviation from the statistical plan outlined above. However, provision for alternative methods and changes to analyses will be included in the statistical analysis plan as specified in the PC-CTU's SOP ST01.01 "Statistical Analysis Plan".

11.8. Qualitative Interviews and Analysis

We will recruit a maximum variation sample of 35 participants across both study arms for the nested qualitative study, continuing recruitment until data saturation is reached. The qualitative researcher will be unblinded to support appropriate participant selection. The topic guide will include participants' experiences and perceptions of recruitment to, and participation in a study requiring a daily treatment (whether D-mannose or placebo), exploring the level of benefit required to continue this type of regime and facilitators and barriers to prophylactic treatment. For participants' convenience, interviews will be conducted by telephone. Thematic analysis of the interviews will take into account issues identified from the literature and clinical research context, as well as inductively allowing new themes and ideas to emerge from the data. Analysis will be guided by the constant comparative method, which will include reading and familiarisation with the transcripts, noting and recording initial themes and then conducting systematic and detailed open coding using NVivo, a qualitative data analysis software. Analysis will proceed in an iterative manner – thus, the coding of a first set of interviews will generate an initial coding framework, which will be further developed and refined as further interviews are conducted and analysis proceeds. The researcher will draw on the clinical expertise of the rest of the research team, in developing the coding framework and critically discussing ideas for categories emerging from the data, to ensure trustworthiness. A reflexive journal will assist in interpreting data and forming conclusions.

Transcripts are de-identified to protect the identity of participants, for example, if these documents are be looked at by the wider research team or verbatim quotations are to be used in publication. However, the qualitative researcher would be able to link transcripts back to the original securely stored recordings. There can sometimes be confusion about the actual meaning of the wording on the transcript and it can be necessary to check back for detail about what is actually being conveyed, including by tone of voice, spacing of words etc. This is important as the person analysing the transcripts is not always the person who conducted the interview.

11.9. Health Economics Analysis

A cost-utility analysis (CUA) from a health system perspective with a time horizon of six months will be conducted alongside this study. The primary outcome measure for CUA will be the quality adjusted life years (QALYs). Data collection to facilitate analysis includes resource use and health outcomes. The participant diary will be the main source of resource use. Unit costs associated with resource use items will be obtained from national standards (e.g. NHS reference costs, British National Formulary or PSSRU. Health outcomes will be measured using the 5-level version of the EQ-5D questionnaire (EQ-5D-5L). Participants will be asked to complete EQ-5D-5L at baseline, and on day 1, 3 and 5 in their diary if they develop any symptoms of UTI.

Data analysis will be conducted on an intention-to-treat basis using an incremental approach. Resource use and unit cost will be combined to calculated health care costs for each participant and mean cost for each study arm. EQ-5D-5L utility values will be calculated using the UK-based algorithm. Using the under

© Copyright: The University of Oxford and Oxford University Hospitals NHS Foundation Trust 2016

the curve methods to combine utility values and associated time durations will produce QALYs for each participant and mean QALYs for each study arm during the 24 week study period. Mean differences in costs and QALYs between the study arms will be estimated as incremental cost per QALY gained. Given the fact that antibiotics are currently the mainstay treatment for both acute and recurrent UTIs, the issue of how the cost of antibiotic resistance should be incorporated into economic evaluation will be explored in the analysis.

12. DATA MANAGEMENT

12.1. Source Data

Source documents are where data are first recorded, and from which participants' CRF data are obtained. These include, but are not limited to, medical records (from which medical history and previous and concurrent medication may be summarised into the CRF), clinical and office charts, laboratory and pharmacy records, diaries, microfiches, radiographs, and correspondence.

CRF entries will be considered source data if the CRF is the site of the original recording (e.g. there is no other written or electronic record of data). All documents will be stored safely in a locked cabinet in a locked room. On all study-specific documents, other than the signed consent, the participant will be referred to by the study participant number/code, not by name.

12.2. Access to Data

Direct access will be granted to authorised representatives from the Sponsor, host institution and the regulatory authorities to permit trial-related monitoring, audits and inspections.

12.3. Data Recording and Record Keeping

Data Management will be performed in accordance with PC-CTU Data Management SOPs. Study specific procedures will be outlined in a Data Management Plan (DMP) to ensure that high quality data are produced for statistical analysis. The DMP is reviewed and signed by all applicable parties including the Study Manager and the Study Statistician prior to the first participant being enrolled.

All participants will be consented using electronic or paper consent forms. Sortition will be used for randomisation and also to record eligibility. Sortition is a secure, web-based, system developed in conjunction with the Primary Care Clinical Trials Unit. The system will incorporate data entry and validation rules to reduce data entry errors, and management functions to facilitate auditing and data quality assurance. Data protection requirements will be embedded into the design of the web-based system and enforced by best practice study management procedures. The clinical data manager will oversee the process of electronic data validation and manual listings, sending out data clarification forms when required and following these up until the queries are resolved.

Once the last participants is enrolled, prior to database lock a dataset review will be undertaken by the Information system manager and study statistician. All critical data items are 100% checked against original source data documents to ensure accuracy, an error rate is established across all fields to ensure a consistently accurate dataset.

© Copyright: The University of Oxford and Oxford University Hospitals NHS Foundation Trust 2016

Participants contact information will be collected in paper form and sent to the study team. The contact details will be stored by the study team separately from all other study data.

We will seek consent from participants to allow us to keep their contact details on record so that we can invite them to take part in future research related to UTIs at the University of Oxford. Participants can decline to give consent for this and still be eligible for the main study. If they do not consent, these details will be de-identified as soon as the required study contact has been completed.

Consent will be sought for the qualitative recordings to be stored securely for five years.

The study team will preserve the confidentiality of all data obtained which are to be kept by the D-mannose study team in compliance with the Data Protection Act (DPA) 2018, the General Data Protection Regulation (GDPR), and PC-CTU Data Management SOP, this includes data of study participants.

13. QUALITY ASSURANCE PROCEDURES

The trial will be conducted in accordance with the current approved protocol, GCP, relevant regulations and standard operating procedures.

Monitoring will be performed according to the study risk assessment. This will be completed before the study starts recruitment and will be reviewed throughout the study.

A trial steering committee will provide oversight of the study, meeting before the study opens to recruitment and within six months of recruitment commencing.

14. SERIOUS BREACHES

The Medicines for Human Use (Clinical Trials) Regulations contain a requirement for the notification of "serious breaches" to the MHRA within 7 days of the Sponsor becoming aware of the breach.

A serious breach is defined as “A breach of GCP or the trial protocol which is likely to affect to a significant degree –

(a) the safety or physical or mental integrity of the subjects of the trial; or

(b) the scientific value of the trial”.

In the event that a serious breach is suspected the Sponsor must be contacted within 1 working day. In collaboration with the C.I, the serious breach will be reviewed by the Sponsor and, if appropriate, the Sponsor will report it to the REC committee, Regulatory authority and the NHS host organisation within seven calendar days.

15. ETHICAL AND REGULATORY CONSIDERATIONS

15.1. Declaration of Helsinki

The Investigator will ensure that this trial is conducted in accordance with the principles of the Declaration of Helsinki.

© Copyright: The University of Oxford and Oxford University Hospitals NHS Foundation Trust 2016

15.2. Guidelines for Good Clinical Practice

The Investigator will ensure that this trial is conducted in accordance with relevant regulations and with Good Clinical Practice.

15.3. Approvals

The protocol, informed consent form, participant information sheet and any proposed advertising material will be submitted to an appropriate Research Ethics Committee (REC), HRA (where required), regulatory authorities (MHRA in the UK), and host institution(s) for written approval.

The Investigator will submit and, where necessary, obtain approval from the above parties for all substantial amendments to the original approved documents.

15.4. Reporting

The CI shall submit once a year throughout the clinical trial, or on request, an Annual Progress Report to the REC, HRA (where required), host organisation and Sponsor. In addition, an End of Trial notification and final report will be submitted to the MHRA, the REC, host organisation and Sponsor.

15.5. Participant Confidentiality

The trial staff will ensure that the participants' anonymity is maintained and a unique ID number will be used to identify participants. There will also be a secure trial management system where participants' identifiable information will be stored in order for the study team to contact participants throughout the trial for all follow-up assessments (weekly texts and monthly phone calls). This data will be destroyed no later than 3 months after the end of the trial. Participants' identifiable data and participant study data will be kept in separate databases throughout and will only be accessible by trial staff and authorised personnel. Any paper documents will be stored securely and only accessible by trial staff and authorised personnel. The trial will comply with the GDPR and DPA 2018, which requires data to be de-identified as soon as it is practical to do so. Details from participants who have consented to be contacted for future research will be kept for a maximum of five years after the end of the trial separately from the trial data.

15.6. Expenses and Benefits

In order to encourage and thank participants for their continuing participation in the study we will offer a £10 voucher at two, four and six months after study entry. The baseline appointment will be conducted either via their local surgery or by telephone or Skype, and follow up will be by telephone and text message, so travel expenses will not be reimbursed.

15.7. Other Ethical Considerations

This study will require women to potentially take a placebo for 24 weeks however this will be fully explained before study entry, there is equipoise over the benefits of D-mannose, and this period of evaluation is the minimum necessary to demonstrate the effectiveness of the intervention.

© Copyright: The University of Oxford and Oxford University Hospitals NHS Foundation Trust 2016

16. FINANCE AND INSURANCE

16.1. Funding

This study is funded by the NIHR School for Primary Care Research.

16.2. Insurance

The University has a specialist insurance policy in place which would operate in the event of any participant suffering harm as a result of their involvement in the research (Newline Underwriting Management Ltd, at Lloyd's of London). NHS indemnity operates in respect of the clinical treatment that is provided.

17. PUBLICATION POLICY

The Investigators will be involved in reviewing drafts of the manuscripts, abstracts, press releases and any other publications arising from the study. Authors will acknowledge that the study was funded by School for Primary Care Research, NIHR. Authorship will be determined in accordance with the ICMJE guidelines and other contributors will be acknowledged.

18. DISSEMINATION

Participants can contact the study team for a copy of the summary the results; copies of the summary of the results will also be provided to all participating GP surgeries so participants can collect a copy at their surgery as well.

19. DEVELOPMENT OF A NEW PRODUCT / PROCESS OR THE GENERATION OF INTELLECTUAL PROPERTY

Not applicable

20. ARCHIVING

At the conclusion of the study and after the database has been locked, all essential documents and trial data will be archived for at least five years in accordance with the PC-CTU's Archiving SOPs. The CI is responsible for authorising retrieval and disposal of archived material.

© Copyright: The University of Oxford and Oxford University Hospitals NHS Foundation Trust 2016

21. REFERENCES

1. Butler CC et al. Antibiotic-resistant infections in primary care are symptomatic for longer and increase workload: outcomes for patients with E. coli UTIs. British Journal of General Practice. 2006;56(530):686-92.

2. Little P et al. Effectiveness of five different approaches in management of urinary tract infection: randomised controlled study. BMJ. 2010;340:c199.

3. Stapleton A. Host factors in susceptibility to urinary tract infections. Advances in Bladder Research: Springer; 1999. p. 351-8.

4. Albert X et al. Antibiotics for preventing recurrent urinary tract infection in non-pregnant women. Cochrane Database Syst Rev. 2004;3.

5. Hooton TM, et al. A prospective study of risk factors for symptomatic urinary tract infection in young women. New England journal of medicine. 1996;335(7):468-74.

6. Sanford JP. Urinary tract symptoms and infections. Annual review of medicine. 1975;26(1):485-98.

7. Ikäheimo R et al. Recurrence of urinary tract infection in a primary care setting: analysis of a 1-year follow-up of 179 women. Clin Infect Dis. 1996 Jan;22(1):91-9

8. Butler CC, et al. Incidence, severity, help seeking, and management of uncomplicated urinary tract infection: a population-based survey. Br J Gen Pract. 2015 Oct;65(639):e702-7.

9. Flower A, et al. How women manage recurrent urinary tract infections: an analysis of postings on a popular web forum. BMC Family Practice. 2014;15(1):1-8.

10 Ciani O et al. An Economic Perspective on Urinary Tract Infection: The “Costs of Resignation” Clinical Drug Investigation April 2013, Volume 33, 4, pp 255–261

11. Maki KC et al. Consumption of a cranberry juice beverage lowered the number of clinical urinary tract infection episodes in women with a recent history of urinary tract infection Am J Clin Nutr. 2016 Jun;103(6):1434-42

12. SIGN. Management of suspected bacterial urinary tract infection in adults. 2012.Available from URL: http://www.sign.ac.uk.

13. Harding GK et al. Long-term antimicrobial prophylaxis for recurrent urinary tract infection in women. Review of Infectious Diseases. 1982;4(2):438-43.

14. Car J et al. Recurrent urinary tract infection in women. BMJ. 2003 ;327.

15. Costelloe C et al. Effect of antibiotic prescribing in primary care on antimicrobial resistance in individual patients: systematic review and meta-analysis. BMJ. 2010 May 18;340

16. Bouckaert J et al. Receptor binding studies disclose a novel class of high-affinity inhibitors of the Escherichia coli FimH adhesin Mol Microbiol, 200.

17. Michaels EK et al. Effect of D-mannose and D-glucose on Escherichia coli bacteriuria in rats Urol Res, 1983.

© Copyright: The University of Oxford and Oxford University Hospitals NHS Foundation Trust 2016

18. Etchison JR, Freeze HH. 1997. Enzymatic assay of D-mannose in serum. Clin Biochem 43:533–8.

19. Sharma et al. Mannose metabolism: more than meets the eye. Biochem Biophys Res Commun. 2014 October 17; 453(2): 220–228.

20. Xing Hu et al. d-Mannose: Properties, Production and Applications: An Overview

21. Kranjčec B et al. D-mannose powder for prophylaxis of recurrent urinary tract infections in women: a randomized clinical study. World J Urol. 2014 Feb;32(1):79-84

22. Phé V et al. Open label feasibility study evaluating D-mannose combined with home-based monitoring of suspected urinary tract infections in patients with multiple sclerosis. Neurourol Urodyn. 2016 Nov 4.

23. Porru D et al. Oral D-mannose in recurrent urinary tract infections in women: a pilot study Journal of Clinical Urology, May 2014; vol. 7, 3: 208-213

© Copyright: The University of Oxford and Oxford University Hospitals NHS Foundation Trust 2016

22. APPENDIX A: TRIAL FLOW CHART

Patient Identification

Route 1 – Face to Face

- Database search and GP screen for potential participants
- (Optional: Phone call to potential participant) Invitation pack sent to participant, if interested booked into appointment with RN/GP

OR

- Participant self presents with UTI and if interested booked into appointment with RN/GP

Route 2 - Remote

- Database search and GP screen for potential participants
- (Optional: Phone call to potential participant) Invitation pack sent to potential participant
- Interested participants return EoI to study team

Route 3 - Advert

- Potentially eligible participants see advert and contact study team
- Study information pack sent to potential participant
- Interested participants return EOI to study team

(Routes 2 and 3:) Initial email with link to online form and time and date for appointment sent

Eligibility and Consent

- Electronic eligibility form completed
- Electronic or paper consent form completed
- Electronic baseline CRF completed
- Eligibility is confirmed by their GP; confirmation email is sent

Randomisation

- Participant randomised to D-mannose or placebo
- Participant sent study product and study pack, including sample pots. Participants to provide a urine sample and an optional perineal swab.

Follow up

- Once a week: text / email message with link to compliance and UTI check
- Once a month: contact to complete short CRF if no previous response and arrange additional supplies
- At month 2, 4 and 6, participant is sent £10 voucher
- During UTI Episode: symptom diary completed and urine samples collected
- Completion of final questionnaire over the phone, on paper or online
- GP notes review carried out 26 weeks after study entry

© Copyright: The University of Oxford and Oxford University Hospitals NHS Foundation Trust 2016

23. APPENDIX B: SCHEDULE OF PROCEDURES

Procedures	Participant Contacts									
	For those in Route 1 and 2; mail out group	Telephone call 1*	Telephone call 2* or at GP practice	After telephone call 2 or baseline visit at GP practice	Weekly contact	4-weekly contact	2 month contact	4 month contact	6 month contact	During UTI episode
Eligibility Check by study team		✓								
Eligibility Check by GP	✓			✓+						
Informed consent			✓							
Demographics		✓	✓							
Medical history		✓	✓							
Randomisation				✓						
Dispensing of study product				✓			✓	✓		
Compliance					✓	✓				
Laboratory tests				✓						✓
SAE Assessments										✓
Symptom Diary										✓
£10 Voucher given							✓	✓	✓	

© Copyright: The University of Oxford and Oxford University Hospitals NHS Foundation Trust 2016

*Telephone call 2 can take place at the same time as phone call one if participant completed consent form has been received

+ For those in route 3 recruitment group (recruitment via advert)

© Copyright: The University of Oxford and Oxford University Hospitals NHS Foundation Trust 2016

24. APPENDIX C: AMENDMENT HISTORY

Amendment No.	Protocol Version No.	Date issued	Author(s) of changes	Details of Changes made
1	3.0	13th March 2019	Dr Gail Hayward, Jared Robinson	- Move the urine sample at 2 months to baseline time point; add optional perineal swab sample collection at baseline<br>- Add option to contact participants via email.<br>- Change of Trial Statistician and addition of Trial Manager
2	4.0	15th August 2019	Dr Gail Hayward, Jared Robinson	- Addition of a medication letter template for participants to use in case of taking study product during travel abroad (airport/customs purposes).<br>- Additional question in final questionnaire re whether patients think they were allocated to study product or placebo for qualitative purposes.<br>- Information added to the 6-month letter re availability of published trial results after study closure, and options for patients to find out if they were taking study product or placebo during the trial.<br>- Reduction in number of UTI episodes collected in the baseline questionnaire regarding the number of UTI episodes and symptoms experienced in previous year to improve data quality.<br>- Clarification of study duration from 6 months to 24 weeks in Protocol.<br>- Minor corrections to wording in Monthly Contact CRF

© Copyright: The University of Oxford and Oxford University Hospitals NHS Foundation Trust 2016

3	5.0	07th November 2019	Dr Gail Hayward, Dr Marloes Franssen	- Addition of extra participants to the qualitative part of the study (changing the number to 35)<br>- Revision of the maximum number of potential recruits from 508 to 598 in response to a greater than estimated withdrawal rate<br>- There will be a secondary analysis based on per protocol population<br>- SAE reporting has been further defined, elective procedures will not be reported
4 (Non-substantial)	6.0	17th July 2020	Dr Gail Hayward, Jared Robinson	- Extension to Study End Date following approval of a no-cost extension by the funder to allow additional time for closeout and final data analysis.<br>- Correction and clarification to time points of objectives and outcome measures.<br>- Addition of Investigator to Protocol

Protocol amendments must be submitted to the Sponsor for approval prior to submission to the REC committee or MHRA.

© Copyright: The University of Oxford and Oxford University Hospitals NHS Foundation Trust 2016
