# Supplementary material for: d-Mannose for Prevention of Recurrent Urinary Tract Infection Among Women: A Randomized Clinical Trial
Source: JAMA Intern Med. 2024 Apr 8;184(6):619–28. doi: 10.1001/jamainternmed.2024.0264 (PMC11002776; doi:10.1001/jamainternmed.2024.0264)

Primary Care Clinical Trials Unit

STATISTICAL ANALYSIS PLAN

merit

MERIT: d-MannosE to prevent Recurrent urine InfecTions

D-Mannose to prevent Recurrent UTI: a double blind randomised placebo-controlled trial

Version 1.0

15 February 2021

	NAME	TITLE	SIGNATURE	DATE
Written by:	Sam Mort	Trial Statistician	[signature]	15 Feb2021
Reviewed by:	Jill Mollison	Senior Trial Statistician	[signature]	23 Feb 2021
Approved by:	Gail Hayward	Chief Investigator	[signature]	15 Feb2021

Version History

Version:	Version Date:	Changes:
1.0	15 February 2021	Original version

TABLE OF CONTENTS

1 INTRODUCTION 4

1.1 PREFACE 4
1.2 PURPOSE AND SCOPE OF THE PLAN 4
1.3 TRIAL OVERVIEW 5
1.4 OBJECTIVES 6
1.4.1 Primary objective 6
1.4.2 Secondary objectives 6
1.4.3 Tertiary objectives 7

2 TRIAL DESIGN 7

2.1 OUTCOME MEASURES 7
2.1.1 Proportion of women experiencing at least one further episode of a clinically suspected UTI 7
2.1.2 Number of days of moderately bad (or worse) symptoms of UTI 7
2.1.3 Time to next consultation with clinically suspected UTI 8
2.1.4 Number of clinically suspected UTIs 8
2.1.5 Number of microbiologically proven UTIs 8
2.1.6 Number of antibiotic courses for UTI 8
2.1.7 Number of days of antibiotic consumption for UTI 9
2.1.8 Defined daily dose (DDD) 9
2.1.9 Total mg by antibiotic type 9
2.1.10 Report of consumption of antibiotics 9
2.1.11 Incidence of antibiotic resistant UTIs 9
2.1.12 Hospital admissions related to UTI 10
*2.1.13 Antibiotic usage .... **Error! Bookmark not defined.***
*2.1.14 Urine culture results for samples sent .... **Error! Bookmark not defined.***
2.2 TARGET POPULATION 10
2.2.1 Inclusion criteria 10
2.2.2 Exclusion criteria 10
2.3 SAMPLE SIZE 10
2.4 RANDOMISATION AND BLINDING IN THE ANALYSIS STAGE 11

3 ANALYSIS – GENERAL CONSIDERATIONS 11

3.1 DESCRIPTIVE STATISTICS 11
3.2 CHARACTERISTICS OF PARTICIPANTS 11
3.3 DEFINITION OF POPULATION FOR ANALYSIS 12
3.4 POOLING OF INVESTIGATIONAL SITES 12
3.5 DATA MONITORING COMMITTEE AND INTERIM ANALYSES 12

4 PRIMARY ANALYSIS 12

4.1 PRIMARY OBJECTIVE: EFFECTIVENESS OF DAILY USE OF D-MANNOSE 12
4.2 HANDLING MISSING DATA 13
4.3 HANDLING OUTLIERS 13
4.4 MULTIPLE COMPARISONS AND MULTIPLICITY 13
4.5 MODEL ASSUMPTIONS 13

5 SECONDARY ANALYSIS 14

5.1 PRIMARY OBJECTIVE: PROPORTION OF WOMEN EXPERIENCING AT LEAST ONE FURTHER EPISODE OF CLINICALLY SUSPECTED UTI 14
5.1.1 Experiencing at least one further episode of clinically suspected UTI ... 14
5.2 SECONDARY OBJECTIVE: SYMPTOM BURDEN OF UTI ... 14
5.2.1 Number of days of moderately bad (or worse) symptoms of UTI ... 14
5.2.2 Time to next consultation with clinically suspected UTI ... 14
5.2.3 Number of clinically suspected UTIs ... 15
5.2.4 Number of microbiologically proven UTIs ... 15
5.3 SECONDARY OBJECTIVE: ANTIBIOTIC PRESCRIPTION FOR UTI ... 15
5.3.1 Number of antibiotic courses for UTI ... 15
*5.3.2 Number of days of antibiotic consumption for UTI ... **Error! Bookmark not defined.***
5.3.3 Defined daily dose (DDD) ... 16
5.3.4 Total mg by antibiotic type ... 16
5.3.5 Report of consumption of antibiotics ... 16
5.4 SECONDARY OBJECTIVE: ANTIBIOTIC RESISTANT UTIS ... 16
5.4.1 Incidence of antibiotic resistant UTIs ... 17
5.5 SECONDARY OBJECTIVE: HOSPITAL ADMISSIONS RELATED TO UTIS ... 17
5.5.1 Hospital admissions related to UTIs ... 17

6 TERTIARY ANALYSIS ... ERROR! BOOKMARK NOT DEFINED.

6.1 TERTIARY OBJECTIVE: ASSOCIATION BETWEEN PREVIOUS ANTIBIOTIC PRESCRIBED AND PRESENCE OF BACTERIA RESISTANT TO ANTIBIOTICS IN UTI ... **ERROR! BOOKMARK NOT DEFINED.**
*6.1.1 Antibiotic usage ... **Error! Bookmark not defined.***
*6.1.2 Urine culture results for samples sent ... **Error! Bookmark not defined.***

7 SENSITIVITY ANALYSIS ... 18

7.1 PER-PROTOCOL ... 18
7.2 FACTORS THAT PREDICT MISSINGNESS ... 18
7.3 IMPUTING MISSING PRIMARY OUTCOME ... 19
7.4 MULTIPLE IMPUTATION ... 19
7.5 WEEKLY SYMPTOM BURDEN OF UTI ... 19

8 SUBGROUP ANALYSES ... 19

9 SAFETY ANALYSIS ... 20

10 EXPLORATORY ANALYSIS ... 20

11 VALIDATION ... 20

12 CHANGES TO THE PROTOCOL OR PREVIOUS VERSIONS OF SAP ... 20

13 REFERENCES ... 21

14 APPENDICES ... 23

1 INTRODUCTION

Trial title: D-mannose to prevent Recurrent UTI: a double blind randomised placebo-controlled trial

Short title: MERIT: d-MannosE to prevent Recurrent urine InfecTions

Ethics Ref: 18/SW/0245

IRAS no.: 245539

This Statistical Analysis Plan (SAP) supports version 6.0 of the protocol dated 17 July 2020

1.1 PREFACE

Chief Investigator: Dr Gail Hayward - gail.hayward@phc.ox.ac.uk

Clinical Trial Manager: Jared Robinson – jared.robinson@phc.ox.ac.uk

Trial Statistician: Nicola Williams – nicola.williams@phc.ox.ac.uk

Lead Statistician: Ly-Mee Yu – ly-mee.yu@phc.ox.ac.uk

Data Manager: Jenna Grabey – jenna.grabey@phc.ox.ac.uk

1.2 PURPOSE AND SCOPE OF THE PLAN

This document details the proposed analysis of the main paper reporting results from the NIHR School for Primary Care research funded randomised controlled trial to assess the effectiveness of D-mannose in preventing symptomatic UTIs. The results reported in these papers should follow the strategy set out here. Subsequent analyses of a more exploratory nature will not be bound by this strategy, though they are expected to follow the broad principles laid down here. The principles are not intended to curtail exploratory analysis (for example, to decide cut-points for categorisation of continuous variables), nor to prohibit accepted practices (for example, data transformation prior to analysis), but they are intended to establish the rules that will be followed, as closely as possible, when analysing and reporting the trial.

The analysis strategy will be available on request when the principal papers are submitted for publication in a journal. Suggestions for subsequent analyses by journal editors or referees, will be considered carefully, and carried out as far as possible in line with the principles of this analysis strategy; if reported, the source of the suggestion will be acknowledged.

It has not been anticipated that there will be any deviations from the statistical plan outlined in this document. However, provisions for alternative methods and changes to analyses will be included in this document as specified in the PC-CTU's SOP ST101 "Statistical Analysis Plan". Any deviations from this statistical analysis plan will be described and justified in the final report of the trial. The analysis should be carried out by an identified, appropriately qualified, and experienced statistician, who should ensure the integrity of the data during their processing. Examples of such procedures include quality control and evaluation procedures.

1.3 Trial overview

Urinary tract infections (UTIs) are the most common bacterial infection seen in women presenting to UK primary care (Butler 2006, Little 2010), accounting for up to 3% of all GP consultations (Stapleton 1999). Approximately 40-50% of women experience one episode during their lives (Stapleton 1999). Recurrent UTIs (RUTIs) are commonly defined in the literature as three episodes of UTIs in the last twelve months or two episodes in the last six months (Albert 2004). Between 20-44% of women who have had one episode of UTI will have a further UTI and around 25% of these will develop subsequent recurrent episodes (Sanford 1975, Hooton 1996, Ikäheimo 1996). In a recent survey of 2424 randomly selected UK women, 3% reported RUTI in the past year, equivalent to 800,000 women annually in the UK (Butler 2015). RUTIs have a significant negative impact on quality of life which extends beyond the unpleasant symptomatology into distressing and disrupted sexual relationships, persistent unmanageable pain and systemic illness (Flower 2014). They have a high impact on health care costs as result of outpatient visits, diagnostic tests and prescriptions (Butler 2015). A recent Italian study estimated a direct costs per episode of RUTI at 142 euros (approximately £125) (Ciani 2013).

To date, antibiotic prophylaxis is the only treatment which has been demonstrated to be beneficial for RUTIs in a systematic review of randomised controlled trials, although a recent large study suggests some benefit from cranberry juice (Maki 2016). Antibiotics given continuously for six to twelve months were better than placebo at preventing recurrent infection (NNT 1.84) (Albert 2004), and national guidelines advocate their use (SIGN, 2012). However, antibiotics also resulted in more severe and unpleasant side effects (e.g. vomiting, urticarial, candidiasis). Furthermore, once prophylaxis is discontinued, even after extended periods, approximately 50-60% of women will become re-infected within three months (Harding 1982, Car 2003). Thus, antibiotic prophylaxis does not exert a long-term effect on the baseline infection rate and antibiotic use is directly linked to antibiotic resistance in uropathogens (Costelloe 2010). Antibiotic resistance has been positively associated with an increased duration of severe symptoms of UTIs, irrespective of the use of an appropriate antibiotic (Costelloe 2010, Little 2010).

D-mannose is a type of sugar (a monosaccharide isomer of glucose) which is considered to inhibit bacterial adherence to uroepithelial cells by binding to a site on the tip of the fimbria (Bouckaert 2005) and has shown benefit in animal models (Michaels 2019). It is an essential glyconutrient for human health. It is an important metabolic intermediate product in the biosynthesis of most secretory proteins and glycoproteins in the human body (Etchison 1997). It is absorbed in the upper gastrointestinal tract and excreted in the urine, and this proposed daily dose is equivalent to less than 2 grams of glucose (Sharma 2014), comparing favourable to cranberry juice which contains 22 grams of sugar per 200ml. It can be found naturally within the diet occurring in numerous food sources, such as coffee, baker’s yeast, egg white, fruits such as apples, cranberries, and mangos, and also in legumes such as soybeans, kidney beans, and peanuts (Hu 2016).

Currently D-mannose is available as food supplement which is favoured by women who have RUTIs, but until recently has had little empirical evidence to support its use. An open label randomised three arm trial including 308 women with RUTI seen in outpatient settings (Sharma 2014) showed that daily use of D-mannose for six months resulted in an absolute reduction in incidence of further urinary tract infection of 45% from a rate of 60% in the usual care arm, with no adverse events. Incidence was reduced by 11% compared to daily antibiotic use. This finding is supported by two recent smaller studies. A feasibility study in 20 women with multiple sclerosis found a reduction in monthly UTI rate with D-mannose use. Over 80% of participants wished to continue using the product beyond the study period (Hu 2016). A small cross-over pilot study in 60 women presenting to urology outpatient clinics found a longer duration to relapse (52 v 200 days) in those taking D-mannose (Kranjčec 2014).

Although there are early indicators of efficacy, the only large study to date (Sharma 2014) was not placebo controlled and had very high URI incidence in the control arm. Furthermore, a microbiologically confirmed UTI

was a requirement for entry to the study, and participants were withdrawn once they developed a UTI on treatment, meaning true incidence of UTI could not be established, a very important measure for women who experience very frequent UTIs, who are also the most likely candidates for prophylaxis. Finally all women on hormonal contraception were excluded, which reduces applicability to the UK female population at highest risk.

D-mannose has the potential to offer valuable alternative to antibiotic prophylaxis in women who experience RUTI and in turn contribute to better antimicrobial stewardship in primary care. It has been suggested to us as a key target for a clinical trial by the patients who suffer with this condition, some of whom find it highly beneficial and all of whom would welcome a high-quality addition to evidence base for this product. This high costs (at least £25 a month) associated with its purchase add weight to the need to establish whether this is treatment which GPs should be advising their patients to pursue.

In order to ensure that women taking part in the trial are receiving a sufficient amount of D-mannose for its proper investigation without requiring alteration to their normal diet we will provide a standardised amount of D-mannose powder; however due to the varying density of the study product this may vary between 1.5 and 2.5 grams. This study will evaluate the efficacy of D-mannose in women suffering with RUTI presenting to UK primary care and its cost effectiveness.

1.4 Objectives

All the study objectives are described below. A full summary of the study objectives and outcome measures can be found in Appendix I.

1.4.1 Primary objective

1) To assess the effectiveness of daily use of D-mannose compared with placebo in preventing symptomatic UTI in women.

1.4.2 Secondary objectives

1) To determine whether daily use of D-mannose compared with placebo by women who experience RUTIs reduces the symptom burden of UTI.
2) To determine whether daily use of D-mannose compared with placebo by women who experience RUTIs reduces antibiotic prescriptions of UTI.
3) To determine whether daily use of D-mannose compared with placebo by women who experience RUTIs can reduce incidence of antibiotic resistant UTIs.
4) To determine the effect of daily use of D-mannose compared with placebo by women who experience RUTIs on hospital admissions related to UTIs.
5) To determine whether daily use of D-mannose compared with placebo by women who experience RUTIs is cost effective.*
6) To determine whether daily use of D-mannose compared with placebo by women who experience RUTIs is considered acceptable and worthwhile by participants*

*These objectives will be analysed as part of the economic and process evaluation and will be reported separately from the main statistical analysis report.

1.4.3 Tertiary objectives

1) To understand the association between previous antibiotic prescribed and presence of bacteria resistant to antibiotics in urinary tract infections in women who experience recurrent UTI.
2) To understand in more detail the microbiology of RUTI, including whether women may carry bacteria in their bladders when asymptomatic and the impact of D-mannose. This is an additional exploratory analysis dependent on further funding and will not be detailed further in this analysis plan.

The tertiary objectives are beyond the scope of this analysis plan and will not be detailed here.

2 Trial design

This is a two arm, individually randomised, double blind placebo controlled, pragmatic trail, evaluating a complex strategy which involves advice to take a food supplement according to the manufacturers advice. The trial approximates the intervention as closely as possible to what would occur in the real world. Participants were randomised 1:1 and were enrolled for 24 weeks. After completion, notes reviews were completed by a GP or nurse to collect primary and secondary outcome data. A subgroup of women from both trial arms were interviewed after they had completed the main trial and finished taking the study product.

See Appendix II for study flowchart.

2.1 Outcome measures

A summary of the study objectives can be found in section 1.4. An outline of the trial procedures and time points can be found in Appendix III. Only the outcomes which pertain to this statistical analysis plan will be listed here.

2.1.1 Proportion of women experiencing at least one further episode of a clinically suspected UTI

The primary objective is to assess the effectiveness of daily use of D-mannose compared with placebo in preventing symptomatic UTI in women. This objective was measured by the proportion of women experiencing at least one further episode of clinically suspected UTI for which they contact ambulatory care (e.g. Out of hour (OOH) primary care, in hours primary care, ambulance, or A&E) within 26 weeks of study entry. This outcome measure was evaluated 26 weeks after study entry at notes review, conducted no sooner than 26 weeks after study entry to allow medical records to be updated and ensure data completeness.

The primary outcome will be derived from the notes review question *"Is there a record of a suspected UTI in a contact with ambulatory care (e.g. in hours primary care, out of hours primary care, minor injury unit or walk in centre, ambulance or Emergency Department) during the six months after study entry? (If not documented, record as 'No')* (Variable *UTIYN_NR*). Whether a participant experiences at least one further episode of a clinically suspected UTI will be marked as affirmative if this question is marked as affirmative.

2.1.2 Number of days of moderately bad (or worse) symptoms of UTI

This outcome will be derived from the participant completed UTI diary question *"Please rate your symptoms today"* for each of the UTI symptoms listed; fever, burning or pain when passing urine, urgency (having to go in a hurry), day time frequency (having to go more often than usual in the day), night time frequency (going more often than usual at night), can't do usual activities, feeling generally unwell, nausea, loin pain (Variables

FEVER_DI, BURNING_DI, URGENCY_DI, DAYFR_DI, NIGHTFR_DI, ACTIVITIES_DI, UNWELL_DI, NAUSEA_DI, LOINPAIN_DI). This outcome will be calculated by counting the number of days a participant has scored their symptom severity as 'moderately bad' (score = 3), 'bad' (score = 4), 'very bad' (score = 5), or 'as bad as it could be' (score = 6) to any of the symptoms listed above on the UTI diary throughout the study period.

If a participant has not completed any UTI diaries this outcome will be missing.

A sensitivity analysis will explore the weekly burden of symptoms. This outcome will be derived from the weekly contact and will calculated as the number of weeks the participant scored their symptom severity as 'moderately bad', 'bad', 'very bad', or 'as bad as it could be' to any of the symptoms listed on the weekly contact (Variables *FEVER_WC, BURNING_WC, URGENCY_WC, DAYFR_WC, NIGHTFR_WC, ACTIVITIES_WC, UNWELL_WC, NAUSEA_WC, LOINPAIN_WC*).

2.1.3 Time to next consultation with clinically suspected UTI

This outcome will be derived from the notes review question *"How many contacts (ambulatory care) are listed in the notes for this UTI (within the 14 days as described above) Date"* (variable *DATE1_NR*). This outcome will be calculated as the number of days between the date of randomisation and the first date of any healthcare professional seen (GP, practice nurse, doctor/nurse in emergency department, out of hours clinician, or ambulance). Participants who have not been seen by a healthcare professional for a suspected UTI will be censored at 26 weeks from date of randomisation; participants who died will be censored at date of death and participants who withdrew consent from notes review will be censored at their last contact by the study team.

2.1.4 Number of clinically suspected UTIs

This outcome will be derived from the notes review question "*If yes, how many suspected UTIs were there in the six months after study entry?*" (Variable *UTI_NR*). New UTIs have been recorded based on the following definition in the notes review "*Any two contacts **less than** 14 days from one another should be classified as **the same episode of** UTI unless it is specifically stated it is a <u>new</u> UTI; any two contacts with **more than** 14 days in between them should be classified as <u>separate episodes of</u> UTI, unless your review of the clinical records contradicts this, for example if it is specifically stated that it is not regarding a new UTI despite being more than 14 days from the last contact".*

2.1.5 Number of microbiologically proven UTIs

This outcome will be derived from the notes review question "*Culture result*" (Variable *RESULT_NR*). Three responses on the notes review are possible: mixed growth, no significant growth, or growth of a particular pathogen. A microbiologically proven UTI is defined as one of the listed particular pathogen (*RESULT_NR =3-17*), or another organism (*RESULT_NR = 18*) if the number of colonies is ≥10^4 (Variable *SPECNUM_NR*).

Mixed growth, no significant growth (*RESULT_NR = 1-2*), and other organisms with <10^4 colonies are not classed as a microbiologically proven UTI.

2.1.6 Number of prescribed antibiotic courses for UTI

This outcome will be derived from the notes review question "*Antibiotics given?"* The total number of times this is recorded as yes across the study period will be calculated for each participant.

2.1.7 Number of days of prescribed antibiotics for UTI

This outcome will be derived from the notes review question "*Antibiotics given? If yes duration in days". The total number of days of antibiotic consumption will be calculated for each participant as the sum of the number of days of each course of antibiotics.*

2.1.8 Defined daily dose (DDD)

This outcome will be derived from the notes review question "*Antibiotics given? If yes: Antibiotic name*" (Variable *ATBTRT_NR*). Each participants defined daily dose will be calculated as per the WHO's instructions (https://www.whocc.no/ddd/definition_and_general_considera/)

2.1.9 Total mg by antibiotic type

This outcome will be derived from the notes review question "*Antibiotics given? If yes: Antibiotic name"* (Variable *ATBTRT_NR*). The total dose (mg) that a participant took of each drug throughout the 26 week study period will be calculated separately (Nitrofurantoin, Trimethoprim, Pivmecillinam, Ciprofloxacin, Co-amoxiclav, Amoxicillin, and Cephalexin). This will be reported descriptively only.

2.1.10 Report of consumption of antibiotics

This outcome will be derived from the participant's UTI symptom diary question "*Have you taken any antibiotics for your symptoms since you last completed this diary?"* (Variable *TAKENABX_DI*). This outcome will be calculated as a binary variable which will be classed as an affirmative response if the participant has responded "*Yes, ones I already had at home"* (*TAKENABX_DI* = 1) or "*Yes, ones prescribed by the doctor or nurse for this episode of symptoms*" (*TAKENABX_DI* = 2). This outcome will be classed as a negative response if the participant has responded "*No*" (*TAKENABX_DI* = 0) on all their diaries. This outcome will also be derived as a discrete variable which will be calculated by the count of times (number of days over the 26 weeks) the participant had an affirmative response to this question as detailed above.

This outcome will be missing if the participant is missing this response on all their available UTI symptom diaries.

2.1.11 Incidence of antibiotic resistant UTIs

This outcome will be derived from the notes review question "*Antibiotic sensitivities for the primary organism only (please complete with sensitive 1, resistance 2, intermediate 3, not tested 9)*". Whether a participant had an incidence of antibiotic resistant UTI will be marked as affirmative if the participant has *"intermediate" or "resistance"* response to any of the antibiotics listed below;

Amikacin, Amoxicillin, Aztreonam, Cefalexin, Ceftazidime, Cefriaxone, Ciprofloxacin, Co-Amoxiclav, Colisten sulphate, Ertapenem, Fosfomycin, Gentamicin, Levofloxacin, Meropenem, Nitrofurantoin, Piptaz, Pivmecilliam, Tobramycin, Trimethoprim, Trimeth-sulamethozazole, Other (Variable *AMIK_NR, AMOX_NR, AZTR_NR, CEFA_NR, CIME_NR, CONE_NR, CIPR_NR, COAM_NR, COLI_NR, ERTA_NR, FOSF_NR, GENT_NR, LEVO_NR, MERO_NR, NITR_NR, PIPT_NR, PIVM_NR, TOBR_NR, TRIM_NR, TSUL_NR, SENS_NR*).

A count of the number of resistant infections will also be calculated but will not be analysed, these will be presented descriptively.

2.1.12 Hospital admissions related to UTI

This outcome will be calculated as a count of the number of hospital admissions related to a UTI derived from the notes review question *"Did the participant have an unscheduled visit to hospital (including A&E) for either UTI or non-UTI reasons during the MERIT trial? If yes: was reason for admission related to UTI?"* (Variable *HOSPREAS1_NR – HOSPREAS10_NR*).

The number of admissions will be calculated as well as a binary variable indicating whether a participant had one or more admissions.

2.2 Target population

Female participants presenting to ambulatory care with RUTIs.

2.2.1 Inclusion criteria

- Participant is willing and able to give informed consent for participation in the study.
- Participant is able to comply with study procedures.
- Female, aged 18 years or above.
- Presenting to ambulatory care with symptoms consistent with UTI (search will be based on UTI / suspected UTI / UTI specific antibiotics) three or more times in the last year or two or more times in the last six months.

2.2.2 Exclusion criteria

The participant may not have entered the trial if ANY of the following applied:

- Female participant who is pregnant, lactating or planning pregnancy during the course of the study.
- Formal diagnosis of interstitial cystitis or overactive bladder syndrome.
- Prophylactic antibiotics started in the last three months and unwilling to discontinue, or intention to start during the next six months.
- Currently using D-mannose and unwilling to discontinue for the duration of the study.
- Nursing home resident (residential home residents will not be excluded).
- Catheterised, including intermittent self-catheterisation.
- Use of Uromune (an 'immunostimulant').
- Participant in a research study involving an investigation product in the past twelve weeks.
- Previous participant in this study.

2.3 Sample size

The most recent study to evaluate prophylactic treatment for RUTI in a similar population (Maki 2016) found that 26.6% of women in the control arm experienced a UTI within six months. Discussion with our PPI advisors suggests that in order to commit to daily use of a prophylactic regime they would require evidence of a least 50% reduction in the chance of a further UTI during the period of prophylaxis. To detect this with 90% power and an alpha of 0.05 we would require 203 participants in each arm. This equates to 508 participants if a 20% loss to follow-up is assumed. This sample size is also adequate to power the key secondary outcome (the number of UTI's experienced over 26 weeks), and detect a relative incidence rate of 0.5 between the treatment and placebo groups, assuming a base rate of 0.36 as estimated by Maki et al (2016). If the estimated percentage of participants who have either withdrawn or failed to respond to any study team communication for an extended

period seems likely to rise above the 20% initially allowed for, we will recruit additional participants, up to maximum of 598 participants.

2.4 Randomisation and blinding in the analysis stage

Randomisation was performed using Sortition, Primary Care Clinical Trial Unit's (PC-CTU) in-house online randomisation system, according to the current version of the standard operating procedure (SOP) PC-CTU SOP IT104 "Randomisation (Sortition)". Block randomisation was implemented with varying block sizes. Randomisation was stratified by practice ensuring a balance of the two arms within each practice. Participants will be randomised to receive six months of D-mannose or matched placebo using an allocation ratio of 1:1. Neither the participants nor the recruiter knew to which arm they had been randomised. For patients recruited acutely concealment was maintained to prevent any treatment decision regarding the UTI following knowledge of the treatment allocation.

If the randomisation system was unavailable an emergency randomisation list was held at the PC-CTU and this was used to randomise the participant. This was done according to the current version of PC-CTU SOP ST05 "Randomisation and Blinding Procedures".

Out of hours code-breaking was not required due to the risk level of D-mannose, which is a sugar naturally occurring within the diet. Based on previous study literature and manufacturer literature we did not anticipate any adverse events which would require code breaking. Code breaking was possible, however, during office hours. The study statistician informed the necessary clinical team of the study allocation. As far as possible the rest of the study team remained blinded to the allocation. If the clinical condition of a participant necessitated breaking the code, this was undertaken using the Sortition unbinding process.

The only unblinded member of the team is the study statistician at final analysis. The study statistician remained blinded to treatment allocation at interim analysis and unblinded at final analysis.

3 Analysis – General considerations

3.1 Descriptive statistics

Frequencies (with percentages) for binary and categorical variables and means (with standard deviations), or medians (with lower and upper quartiles), and the range (minimum and maximum values) for continuous variables will be presented.

3.2 Characteristics of participants

The following baseline characteristics of participants will be summarised for both randomised groups. There will be no tests of statistical significance nor confidence intervals for differences between randomised groups on any baseline variable:

- Age
- Medical details including: diagnosis and treatment for urinary incontinence, whether they are pre or post-menopausal, hormonal contraception use and hormone replacement therapy.

- Detailed history of episodes of UTI over the previous year, including those which were not reported to ambulatory care and symptom burden of each episode (i.e. severity of symptoms during episodes).
- Known / suspected risk factors for recruitment UTI e.g. sexual activity.
- Current usage of preventative medications for UTI
- EQ-5D-5L index and VAS

Participant throughput from screening through randomisation, follow-up, and analysis will be presented in a CONSORT flow diagram, and include reasons for withdrawal (see Appendix IV).

3.3 Definition of population for analysis

All eligible randomised participants for whom data is available will be analysed according to the groups they were randomly allocated to, regardless of deviations from the protocol. Non-informative censoring will be assumed for those participants that were withdrawn from the study or lost to follow-up for the primary analysis.

A per-protocol analysis will be conducted where compliance to the protocol will be taken into account. Compliance will be defined as those participants who have taken the study product for at least 4 out of the 7 days a week at least for at least 20 weeks of the study period. A second slightly less stringent per-protocol analysis will also be conducted, compliance in this analysis will be defined as those participants who have taken the study product for at least 3 out of the 7 days a week for at least 15 weeks of the study period.

3.4 Pooling of investigational sites

Randomisation was stratified by recruitment site. Site will be adjusted for in the analysis by including a covariate in the statistical models by means of a random effect. If sites recruited only a small number of participants to the study then these will be group together in an ‘other’ category for the covariate in the model.

3.5 Data Monitoring Committee And Interim Analyses

No futility analysis or interim analysis was planned for this study.

4 PRIMARY ANALYSIS

4.1 Primary objective: Effectiveness of daily use of D-mannose

The primary objective is to assess the effectiveness of daily use of D-mannose compared with placebo in preventing symptomatic UTI in women. This objective was measured by the proportion of women experiencing at least one further episode of clinically suspected UTI for which they contact ambulatory care (Out of hour (OOH) primary care, in hours primary care, ambulance, or A&E) within 26 weeks after study entry. This outcome measure was evaluated 26 weeks after study entry during notes review.

The proportion of women experiencing at least one further episode of clinically suspected UTI for which they contact ambulatory care will be derived as described in section 2.1.1.

The number and percentage of women experiencing at least one further episode of clinically suspected UTI for which they contact ambulatory care within 26 weeks of study entry will be reported for each arm of the study.

A generalised linear mixed effects model with a Binomial distribution and a log link function will be used to analyse the primary outcome, with the binary outcome as the dependent variable. Randomised arm will be included in the model as a fixed effect and practice as a random effect. The adjusted relative risk between the intervention arm and the placebo arm with the associated 95% confidence interval and P-value will be derived from the model.

If the log-Binomial generalised linear mixed model fails to converge a log-Poisson generalised linear mixed model with robust standard errors will be used instead. If the log-Poisson model also fails converge a linear logistic regression will be used including randomised group and practice as covariates in the model and the adjusted relative risk will be calculated from the odds ratio with delta-method standard errors (Norton 2013).

4.2 Handling missing data

Data will be assumed to be missing at random.

Numbers of those randomised, excluded due to ineligibility (with reasons), completion of follow-up assessments, withdrawals, and loss to follow-up at each time point over the study period will be reported for each randomised arm, and for the total number randomised. A comparison between the randomised arms in those who have the primary outcome available and those who do not will be performed using a logistic regression model, the odds ratio and associated 95% confidence interval and P-value will be derived from the model.

The frequency and percentage of participants that have data available for the primary and secondary outcome measures will be summarised by randomised arm.

The generalised linear mixed model implicitly accounts for data missing at random, however the data missing mechanism will be explored. Summary statistics will be presented for baseline covariates of those participants who completed and those who were lost to follow-up for the primary outcome. The missing at random assumption will be tested by analysing each baseline covariate in separate logistic regression model to determine which (if any) are associated with missingness of the primary outcome, the associated P-values will be reported alongside the summary statistics. A sensitivity analysis will be conducted on the primary analysis including any baseline factors that were found to be associated with missingness of the primary outcome. Any changes to the assumptions made in the primary analysis will be considered in a sensitivity analysis.

4.3 Handling outliers

The primary outcome is binary, and comes from the participants notes review at the end of the study, so therefore we do not expect any outlier values.

4.4 Multiple comparisons and multiplicity

This is a two arm trial. The sample size used an alpha value of 0.05 in order to maintain an overall Type I error rate of 5% (2-sided). No adjustment for multiple comparisons is necessary. An effect will be interpreted as significant if the P-value is below 0.05.

4.5 Model assumptions

Model assumptions will be assessed using graphical representations of residuals. If the assumptions of the primary analysis are not satisfied, a non-parametric approach will be applied to the data and the difference in medians and 95% CI will be reported.

5 SECONDARY ANALYSIS

5.1 PRIMARY OBJECTIVE: PROPORTION OF WOMEN EXPERIENCING AT LEAST ONE FURTHER EPISODE OF CLINICALLY SUSPECTED UTI

5.1.1 EXPERIENCING AT LEAST ONE FURTHER EPISODE OF CLINICALLY SUSPECTED UTI

In addition to the adjusted relative risk, the unadjusted risk difference in the risk of experiencing at least one further episode of clinically suspected UTI in the treatment arm versus the placebo arm will be reported with the associated 95% confidence interval and P-value.

5.2 SECONDARY OBJECTIVE: SYMPTOM BURDEN OF UTI

The first secondary objective is to determine whether daily use of D-mannose compared with placebo by women who experience RUTIs reduces the symptom burden of UTIs. This objective was measured by four outcomes;

i) Number of days of moderately bad (or worse) symptoms of UTI throughout the study on the participant's diary
ii) Time to next consultation with clinically suspected UTI
iii) Number of clinically suspected UTIs
iv) Number of microbiologically proven UTIs

Outcomes ii, iii, and iv above were evaluated six months after study entry during notes review.

5.2.1 NUMBER OF DAYS OF MODERATELY BAD (OR WORSE) SYMPTOMS OF UTI

The number of days of moderately bad (or worse) symptoms of UTI outcome will be derived as described in section 2.1.2.

A linear mixed effect model will be used to analyse this outcome, with the number of days of moderately bad (or worst) symptoms of UTI as the dependent variable. Randomised group will be included in the model as a fixed effect and practice will be included as a random effect. The adjusted mean difference between the intervention arm and the placebo arm with the associated 95% confidence interval and P-value will be derived from the model. If the assumptions of the linear mixed effect model are violated, then a non-parametric test, such as a quantile regression will be used.

5.2.2 TIME TO NEXT CONSULTATION WITH CLINICALLY SUSPECTED UTI

The time to next consultation with clinically suspected UTI will be derived as described in section 2.1.3.

A mixed effects Cox proportional hazards model will be used to analyse this outcome, with the time to next consolation with clinically suspected UTI as the dependent variable. Randomised group will be included in the model as a fixed effect and practice will be included as a random effect. The adjusted hazard ratio between the

intervention arm and the placebo arm with the associated 95% confidence interval and P-value will be derived from the model.

5.2.3 Number of clinically suspected UTIs

The number of clinically suspected UTIs will be derived as described in section 2.1.4.

A generalised linear mixed effects model with a Poisson distribution, a log link function, and robust standard errors will be used to analyses this outcome, with the number of clinically suspected UTIs as the dependent variable. Randomised group will be included in the model as a fixed effect and practice will be included as a random effect. The adjusted incidence rate ratio between the intervention arm and the placebo arm with the associated 95% confidence interval and P-value will be derived from the model.

5.2.4 Number of microbiologically proven UTIs

The number of microbiologically proven UTIs will be derived as described in section 2.1.5.

A generalised linear mixed effects model with a Poisson distribution, a log link function, and robust standard errors will be used to analyse this outcome, with the number of microbiologically proven UTIs as the dependent variable. Randomised group will be included in the model as a fixed effect and practice will be included as a random effect. The adjusted incidence rate ratio between the intervention arm and the placebo arm with the associated 95% confidence interval and P-value will be derived from the model.

5.3 Secondary objective: Antibiotic prescription for UTI

The second secondary objective is to determine whether daily use of D-mannose compared with placebo by women who experience RUTIs reduces antibiotic prescriptions for UTI. This objective was measured by four outcomes;

i) The number of antibiotics course for UTI; defined daily dose (DDDs) and total mg by antibiotic type
ii) Report of consumption of antibiotic using dairy during periods of infection

The outcomes above were evaluated at notes review and throughout the study.

5.3.1 Number of prescribed antibiotic courses for UTI

The number of antibiotic courses for UTI will be derived as described in section 2.1.6.

A generalised linear mixed effects model with a Poisson distribution, a log link function, and robust standard errors will be used to analyse this outcome, with the number of antibiotic courses as the dependent variable. Randomised group will be included in the model as a fixed effect and practice will be included as a random effect. The adjusted incidence rate ratio between the intervention arm and the placebo arm with the associated 95% confidence interval and P-value will be derived from the model.

5.3.2 Number of days of prescribed antibiotics for UTI

The number of days of antibiotic consumption for UTI will be derived as described in section 2.1.7.

A linear mixed effect model will be used to analyse this outcome, with the number of days of prescribed antibiotics for UTI as the dependent variable. Randomised group will be included in the model as a fixed effect

and practice will be included as a random effect. The adjusted mean difference between the intervention arm and the placebo arm with the associated 95% confidence interval and P-value will be derived from the model. If the assumptions of the linear mixed effect model are violated, then a non-parametric test, such as a quantile regression will be used.

5.3.3 Defined daily dose (DDD)

The defined daily dose (DDD) will be derived as described in section 2.1.8.

A linear mixed effects model will be used to analyse this outcome, with defined daily dose (DDD) as the dependent variable. Randomised group will be included in the model as fixed effects, and practice as a random effect. The adjusted mean difference between the intervention arm and the placebo arm with the associated 95% confidence interval and P-value will be derived for the model. If the assumptions of the linear mixed effect model are violated, then a non-parametric test, such as a quantile regression will be used. The overall DDD will be analysed between the randomised groups as well as the DDD for each individual antibiotics DDD.

5.3.4 Total mg by antibiotic type

The total mg by antibiotic type will be derived as described in section 2.1.9.

The total mg by antibiotic type will not be analysed statistically, this outcome will be summarised descriptively for each drug type by randomised group only.

5.3.5 Report of consumption of antibiotics

The reporting of consumption of antibiotics outcome will be derived as described in section 2.1.10.

The binary variable for report of consumption of antibiotics (yes/no) will be analysed by means of a generalised linear mixed effects model with a Binomial distribution, and a log link function. Randomised group will be included in the model as a fixed effect and practice will be included as a random effect. The adjusted relative risk between the intervention arm and the placebo arm with the associated 95% confidence interval and P-value will be derived from the model. If the log-Binomial generalised linear mixed model fails to converge a log-Poisson generalised linear mixed model with robust standard errors will be used instead. If the log-Poisson model also fails converge a linear logistic regression will be used including randomised group and practice as covariates in the model and the adjusted relative risk will be calculated from the odds ratio with delta-method standard errors (Norton 2013).

The discrete variable for report of consumption of antibiotics (count of number of antibiotics) will be analysed by means of a generalised linear mixed effects model with a Poisson distribution, a log link function, and robust standard errors. Randomised group will be included in the model as a fixed effect and practice will be included as a random effect. The adjusted incidence rate ratio between the intervention arm and the placebo arm with the associated 95% confidence interval and P-value will be derived from the model.

5.4 Secondary objective: Antibiotic resistant UTIs

The third secondary objective is to determine whether daily use of D-mannose compared with placebo by women who experience RUTIs can reduce incidence of antibiotic resistant UTIs. This objective was measured by the proportion of women with a resistant uropathogen culture during an episode of acute infection. This outcome measure was evaluated six months after study entry during notes review.

5.4.1 Incidence of antibiotic resistant UTIs

The incidence of antibiotic resistant UTIs will be derived as described in section 2.1.11.

A generalised linear mixed effects model with a Binomial distribution, and a log link function will be used to analyse this outcome, with antibiotic resistant UTIs to any antibiotics (yes/no) as the dependent variable. Randomisation group will be included as a fixed effect in the model and practice will be included as a random effect. The adjusted relative risk between the intervention arm and the placebo arm with the associated 95% confidence interval and P-value will be derived from the model. If the log-Binomial generalised linear mixed model fails to converge a log-Poisson generalised linear mixed model with robust standard errors will be used instead. If the log-Poisson model also fails converge a linear logistic regression will be used including randomised group and practice as covariates in the model and the adjusted relative risk will be calculated from the odds ratio with delta-method standard errors (Norton 2013).

Number of antibiotic resistant UTIs and antibiotic resistant UTIs for each antibiotic type will be summarised descriptively only.

5.5 Secondary objective: Hospital admissions related to UTIs

The fourth secondary objective is to determine the effect of daily use of D-mannose compared with placebo by women who experience RUTIs on hospital admissions related to UTI. This objective was measured by hospital admission related to UTI. This outcome measured was evaluated six months after study entry during notes review.

5.5.1 Hospital admissions related to UTIs

Hospital admissions related to UTI will be derived as described in section 2.1.12.

The number of hospital admissions related to UTIs will be analysed by means of a generalised linear mixed effects model with a Poisson distribution, a log link function, and robust standard errors. Randomised group will be included in the model as a fixed effect and practice will be included as a random effect. The adjusted incidence rate ratio between the intervention arm and the placebo arm with the associated 95% confidence interval and P-value will be derived from the model.

The binary variable for hospital admissions related to UTIs will be analysed by means of a generalised linear mixed effects model with a Binomial distribution, and a log link function. Randomised group will be included in the model as a fixed effect and practice will be included as a random effect. The adjusted relative risk between the intervention arm and the placebo arm with the associated 95% confidence interval and P-value will be derived from the model. If the log-Binomial generalised linear mixed model fails to converge a log-Poisson generalised linear mixed model with robust standard errors will be used instead. If the log-Poisson model also fails converge a linear logistic regression will be used including randomised group and practice as covariates in the model and the adjusted relative risk will be calculated from the odds ratio with delta-method standard errors (Norton 2013).

6 SENSITIVITY ANALYSIS

Sensitivity analysis will be conducted with respect to the primary outcome only (unless explicitly stated) and will explore the sensitivity of results to different assumptions regarding missing data, outliers, and departure from the randomisation policy.

6.1 PER-PROTOCOL

A sensitivity analysis will be based on a per-protocol population that will be defined as the subsample of participants who have taken the allocated intervention. A question during the weekly contact asks the participant how many days did they take the study product (variable *daystaken_wc*). Participants will be included in the per-protocol analysis if they have taken the study product for at least 4 out of the 7 days a week for at least 20 weeks of the study period.

A generalised linear mixed effects model with a Binomial distribution, and a log link function, similar to the primary analysis in section 4.1 will be used to reanalyse the primary outcome on just the per-protocol population. Randomised arm will be included in the model as a fixed effect and practice will be included as a random effect. The adjusted relative risk between the intervention arm and the placebo arm with the associated 95% confidence interval and P-value will be derived from the model.

A second per-protocol analysis will be conducted with a slightly less stringent criteria; participants will be included in the second per-protocol analysis if they have taken the study product for at least 3 out of the 7 days a week for at least 15 weeks of the study period.

6.2 COMPLIER AVERAGE CAUSAL EFFECT

Missing values of the primary outcome will be imputed from age, reported sexual activity, and any baseline characteristics predictive of missingness of the primary outcome. To obtain an unbiased estimate of the effect of compliance on treatment effect, we will estimate the complier-average causal effect (CACE) (Dunn et al, 2005). This will allow us to obtain an unbiased estimate of the effectiveness of the intervention in those where uptake of the intervention is considered 'adequate'. Adequate will be defined as having taken the study product for at least 4 out of the 7 days a week for at least 20 weeks of the study period. A second CACE analysis will be conducted with a slightly less stringent criteria for adequate; participants will be included in the second CACE analysis if they have taken the product for at least 3 out of out the 7 days a week for at least 15 weeks of the study period.

6.3 FACTORS THAT PREDICT MISSINGNESS

The generalised linear mixed effects model assumes that the data are missing at random (MAR). A logistic regression analysis, described in section 4.2 will be conducted to investigate factors (if any) that are predictive of non-response of the primary outcome. If any factors are associated with non-response, the generalised linear mixed effects model in section 4.1 will be re-run with these factors included as fixed effects, alongside randomised group as a fixed effect, and practice as a random effect. The adjusted relative risk between the intervention arm and the placebo arm with the associated 95% confidence interval and P-value will be derived from the model.

6.4 Imputing missing primary outcome

Two separate generalised linear mixed effects model with a Poisson distribution, a log link function, and robust standard errors, similar to the primary analysis in section 4.1 will be used to reanalyse the primary outcome with the addition of the participants who are missing their primary outcome by imputing it with each of the alternatives for this variable;

i) Yes – Experienced at least one further episode of clinically suspected UTI
ii) No – Did not experience at least one further episode of clinically suspected UTI

Randomised arm will be included in the models as a fixed effect and practice will be included as a random effect. The adjusted relative risks between the intervention arm and the placebo arm with the associated 95% confidence intervals and P-values will be derived from the models.

6.5 Multiple imputation

Participants with missing primary outcome will have their primary outcome imputed using multiple imputation (MI). The MI will be conducted with age, reported sexual activity, and any baseline characteristics predictive of missingness of the primary outcome. The generalised linear mixed model in section 4.1 will be re-run with the imputed missing outcome. The adjusted relative risks between the intervention arm and the placebo arm with the associated 95% confidence intervals and P-values will be derived from the models. This sensitivity analysis will only be conducted if more than 10% of the primary outcome is missing.

6.6 Weekly symptom burden of UTI

The linear mixed model in section 5.2.1 will be re-run with the number of weeks of moderately bad (or worst) symptoms of UTI as the dependent variable. The adjusted mean difference between the intervention arm and the placebo arm with the associated 95% confidence intervals and P-values will be derived from the model.

7 SUBGROUP ANALYSES

No subgroup analysis was planned for in the protocol; however, it was suggested by investigators to include two subgroup analyses. The subgroup analyses will be conducted the same as for the primary analysis, including an additional fixed effect for the categorical subgroup variable and an interaction term for the subgroup variable and randomised group. The results for the subgroup analysis will be reported in a forest plot, along with the overall intervention effect. In addition to the effect size and 95% confidence interval for the intervention effect in each level of the subgroup, the P-value for the interaction term will also be reported.

The subgroups of interest are:

1) More frequent UTIs at baseline (variable *EP12MON_BL*) dichotomised by the median
2) Pre vs. post menopause (variable *MENOPAUSE_BL*), as post menopause can cause symptoms that mimic UTI.

8 SAFETY ANALYSIS

All participants randomised will be included in the safety analysis.

A serious adverse event (SAE) is any untoward medical occurrence that:

- Results in death
- Is life-threatening
- Requires inpatient hospitalisation or prolongation of existing hospitalisation
- Results in persistent or significant disability / incapacity
- Consists of a congenital anomaly of birth defect.

Other 'important medical events' may also be considered serious if they jeopardise the participant or require an intervention to prevent one of the about consequences.

NOTE: The term "life-threatening" in the definition of "serious" refers to an event in which the participant was at risk of death at the time of the event; it does not refer to an event which hypothetically might have caused death if it were more severe.

All SAEs occurring during the trial period shall be detailed containing the following information, description of serious adverse event, start data, end date, severity, reason event is classed as serious, outcome, event related to intervention, allocated intervention arm (variables *SAETERM_SAE, SAESTDAT_SAE, SAEENDAT_SAE, SAESEV_SAE, SAEDTH_SAE, SAELIFE_SAE, SAEHOSP_SAE, SAECONG_SAE, SAEDISAB_SAE, SAEMIE_SAE, SAEOUT_SAE, SAEREL_SAE*). As the numbers are expected to be very low no statistical comparison tests will be performed.

As D-mannose is a food supplement and found naturally within the diet, non-serious adverse events were not collected.

9 EXPLORATORY ANALYSIS

A question on the final questionnaire asks the participant do they think they were taking the study product or the placebo during their participation in the trial. An exploratory analysis will be conducted by means of a logistic regression to explore if the participants were correct and if they knew whether they were taking study product or placebo.

10 VALIDATION

At a minimum the primary analysis, sensitivity analysis, and safety analysis in the statistical analysis report will be validated. Validation will be conducted by a trial statistician who has not performed the main analysis or authored the SAP.

11 CHANGES TO THE PROTOCOL OR PREVIOUS VERSIONS OF SAP

A CACE analysis has been included to the sensitivity analysis section, this was not mentioned in the protocol and was added at the request of the chair of the TSC prior to data lock.

12 REFERENCES

Albert X, Huertas I, Pereiró II, Sanfélix J, Gosalbes V, Perrota C. Antibiotics for preventing recurrent urinary tract infection in non-pregnant women. *The Cochrane Database of Systematic Reviews* 2004;(3):CD001209. [DOI: 10.1002/14651858.CD001209.pub2; PUBMED: 15266443]

Bouckaert J, Berglund J, Schembri M, De Genst E, Cools L, Wuhrer M, Hung CS, Pinkner J, Slättegård R, Zavialov A, Choudhurry D, Langermann S, Hultgren SJ, Wyns L, Klemm P, Oscarson S, Knight SD, De Greve H. Receptor binding studies disclose a novel class of high-affinity inhibitors of the Escherichia coli FimH adhesion. *Molecular Microbiology* 2005;**55**(2):441-455. [DOI: 10.1111/j.1365-2958.2004.04415.x; PUBMED: 15659162]

Butler CC, Hawking MK, Quigley A, McNulty CA. Incidence, severity, help seeking, and management of uncomplicated urinary tract infection: a population-based survey. *The British Journal of General Practice* 2015;**65**(639):e702-707. [DOI: 10.3399/BJGP15x686965; PUBMED: 26412847]

Butler CC, Hillier S, Roberts Z, Dunstan F, Howard A, Palmer S. Antibiotic-resistant infections in primary care are symptomatic for longer and increase workload: outcome for patients with E. coli UTIs. *The British Journal of General Practice* 2006;**56**(530):682-692. [PUBMED: 16954001]

Car J, Sheikh A. Recurrent urinary tract infection in women. *BMJ* 2003;**327**. [DOI: 10.1136/bmj.327.7425.1204]

Ciani O, Grassi B, Tarricone R. An economic perspective on urinary tract infections: the "costs of resignation". *Clinical Drug Investigation* 2013;**33**(4):255-261. [DOI: 10.1007/s40261-013-0069-x; PUBMED: 23475540]

Costelloe C, Metcalfe C, Lovering A, Mant D, Hay AD. Effect of antibiotic prescribing in primary care on antimicrobial resistance in individual patients: systematic review and meta-analysis. *BMJ* 2010;**340**. [DOI: 10.1136/bmj.c2096]

Etchison JR, Freeze HH. Enzymatic assay of D-mannose in serum. *Clinical Chemistry* 1997;**43**(3):533-538. [PUBMED: 9068599]

Flower A, Bishop FL, Lewith G. How women mange recurrent urinary tract infections: an analysis of postings on a popular web forum. *BMC Family Practice* 2014;**15**:162. [DOI: 10.1188/1471-2296-15-162; PUBMED: 25260870]

Harding GK, Ronald AR, Nicolle LE, Thomson MJ, Gray GJ. Long-term antimicrobial prophylaxis for urinary tract infection in women. *Reviews of Infectious Diseases* 1982;**4**(2):438-443. [PUBMED: 6981161]

Hooton TM, Scholes D, Hughes JP, Winter C, Roberts PL, Stapleton AE, Stergachis A, Stamm WE. A prospective study of risk factors for symptomatic urinary tract infection in young women. *The New English Journal of Medicine* 1996;**335**(7):468-474. [DOI: 10.1056/NEJM199608153350703; PUBMED: 9672152]

Norton EC, Miller MM, Kleinman LC. Computing adjusted risk ratios and risk differences in Stata. *The Stata Jounral* 2013;**13**(3):492-509.

Ikäheimo R, Siitonen A, Heiskanen T, Kärkkäinen U, Kuosmanen P, Lipponen P, Mäkelä PH. Recurrence of urinary tract infection in a primary care setting: analysis of a 1-year follow-up of 179 women. *Clinical Infectious Diseases* 1996;**22**(1):91-99. [DOI: 10.1093/clinids/22.1.91; PUBMED: 8824972]

Kranjčec B, Papeš D, Altarac S. D-mannose powder for prophylaxis of recurrent urinary tract infections in women: a randomized clinical trial. *World Journal of Urology* 2014;**32**(1):79-84. [DOI: 10.1007/s00345-013-1091-6; PUBMED: 23633128]

Little P, Moore MV, Turner S, Rumsby K, Warner G, Lowes JA, Smith H, Hawke C, Leydon G, Arscott A, Turner D, Mullee M. Effectiveness of five different approaches in management of urinary tract infection: a randomised controlled trial. *BMJ* 2010;**340**:c199. [DOI: 10.1136/bmj.c199]

Little P, Turner S, Rumsby K, Warner G, Moore M, Lowes JA, Smith H, Hawke C, Turner D, Leydon GM, Arscott A, Mullee M. Dipstick and diagnostic algorithms in urinary tract infection: development and validation, randomised trial, economic analysis, observation cohort, and qualitative study. *Health Technology Assessment* 2009;**13**(19):iii-iv, ix-xi, 1-73. [DOI: 10.3310/hta13190; PUBMED: 19364448]

Maki KC, Kaspar KL, Khoo C, Derrig LH, Schild AL, Gupta K. Consumption of a cranberry juice beverage lowered the number of clinical urinary tract injection episodes in women with a recent history of urinary tract infection. *The American Journal of Clinical Nutrition* 2016;**103**(6):1434-1442. [DOI: 10.3945/ajcn.116.130542; PUBMED: 27251185]

Michaels EK, Chmiel JS, Plotkin BJ, Schaeffer AJ. Effect of D-mannose and D-glucose on Escherichia coli bacteriuria in rats. *Urological Research* 1983;**11**(2):97-102. [PUBMED: 6346629]

Sanford JP. Urinary tract symptoms and infections. *Annual Review of Medicine* 1975:**26**(1):485-498. [DOI: 10.1146/annurev.me.26.020175.002413]

Sharma V, Ichikawa M, Freeze HH. Mannose metabolism: more than meets the eye. *Biochemical and Biophysical Research Communications* 2014;**453**(2):220-228. [DOI: 10.1016/j/bbrc.2014.06.021; PUBMED: 24931670]

SIGN. Management of suspected bacterial urinary tract infection in adults. 2012. Available from URL: http://www.sign.ac.uk.

Stapleton A. Host factors in susceptibility to urinary tract infections. *Advances in Experimental Medicine and Biology* 1999;**462**:351-358. [DOI: 10.1007/978-1-4615-4737-2_27; PUBMED: 10599438]

Hu X, Shi Y, Zhang, P, Miao M, Zhang T, Jiang B. d-Mannose: Properties, Production, and Applications: An Overview. *Comprehensive Reviews in Food Science and Food Safety* 2016;**15**(4):773-785. [DOI: 10.111/1541-4337.12211]

13 APPENDICES

APPENDIX I. OBJECTIVES AND OUTCOME MEASURES

Objectives	Outcome Measures	Timepoint(s) of evaluation of this outcome measure (if applicable)
**Primary Objective**<br>To assess the effectiveness of daily use of D-mannose compared with placebo in preventing symptomatic UTI in women	The proportion of women experiencing at least one further episode of clinically suspected UTI for which they contact ambulatory care (Out of hour (OOH) primary care, in hours primary care, ambulance or A&E) within six months of study entry.	26 weeks after study entry (notes review)
**Secondary Objectives**<br>To determine whether daily use of D-mannose compared with placebo by women who experience RUTIs reduces the symptom burden of UTI	Number of days of moderately bad (or worse) symptoms of UTI (participant dairy)<br><br>Time to next consultation with a clinically suspected UTI<br><br>Number of clinically suspected UTIs<br><br>Number of microbiologically proven UTIs	Throughout study (participant dairy)<br><br>26 weeks after study entry (notes review)
To determine whether daily use of D-mannose compared with placebo by women who experience RUTIs reduces antibiotic prescription for UTI	Number of antibiotic courses for UTI; Defined daily dose (DDDs) and total mg by antibiotic type<br><br>Report of consumption of antibiotics using diary during periods of infection	26 weeks after study entry (notes review)<br><br>Throughout study (participant diary)
To determine whether daily use of D-mannose compared with placebo by women who experience RUTIs reduces antibiotic resistance UTIs	Proportion of women with a resistant uropathogen culture during an episode of acute infection	26 weeks after study entry (notes review)
To determine the effect of daily use of D-mannose compared with placebo by women who experience RUTIs on hospital admissions related to UTI	Hospital admissions related to UTI	26 weeks after study entry (notes review)

To determine whether daily use of D-mannose compared with placebo by women who experience RUTIs is cost effective	Quality of life and healthcare utilisation data collection	Quality of life: baseline, 6 months and during UTI episodes on day 1, 3 and 5 (participant diary)<br><br>Healthcare utilisation: 26 weeks after study entry (notes review)
To determine whether daily use of D-mannose compared with placebo by women who experience RUTIs is considered acceptable and worthwhile by participants	Acceptability and process evaluation conducted via telephone interviews with up to 25 women	Throughout the study
**Tertiary Objectives**<br>To understand the association between previous antibiotic prescribed and presence of bacteria resistant to antibiotics in urinary tract infections in women who experience recurrent UTI	Antibiotic usage in the study period<br><br>Urine culture results for samples sent during the study period	Notes review
To understand in more detail the microbiology of RUTI, including whether women may carry bacteria in their bladders when asymptomatic and the impact of D-mannose. This is an additional exploratory analysis dependent on further funding	Dependent on further funding: Patterns of microbial presence as demonstrated by standard urine culture techniques and next Generation DNA Sequencing in RUTI, exploring association between frequency of infection and microbial presence in the bladder and on the perineum, relationship between asymptomatic and symptomatic microbial presence, and evaluating the impact of D-mannose on microbial presence	Throughout study

APPENDIX II. STUDY FLOWCHART

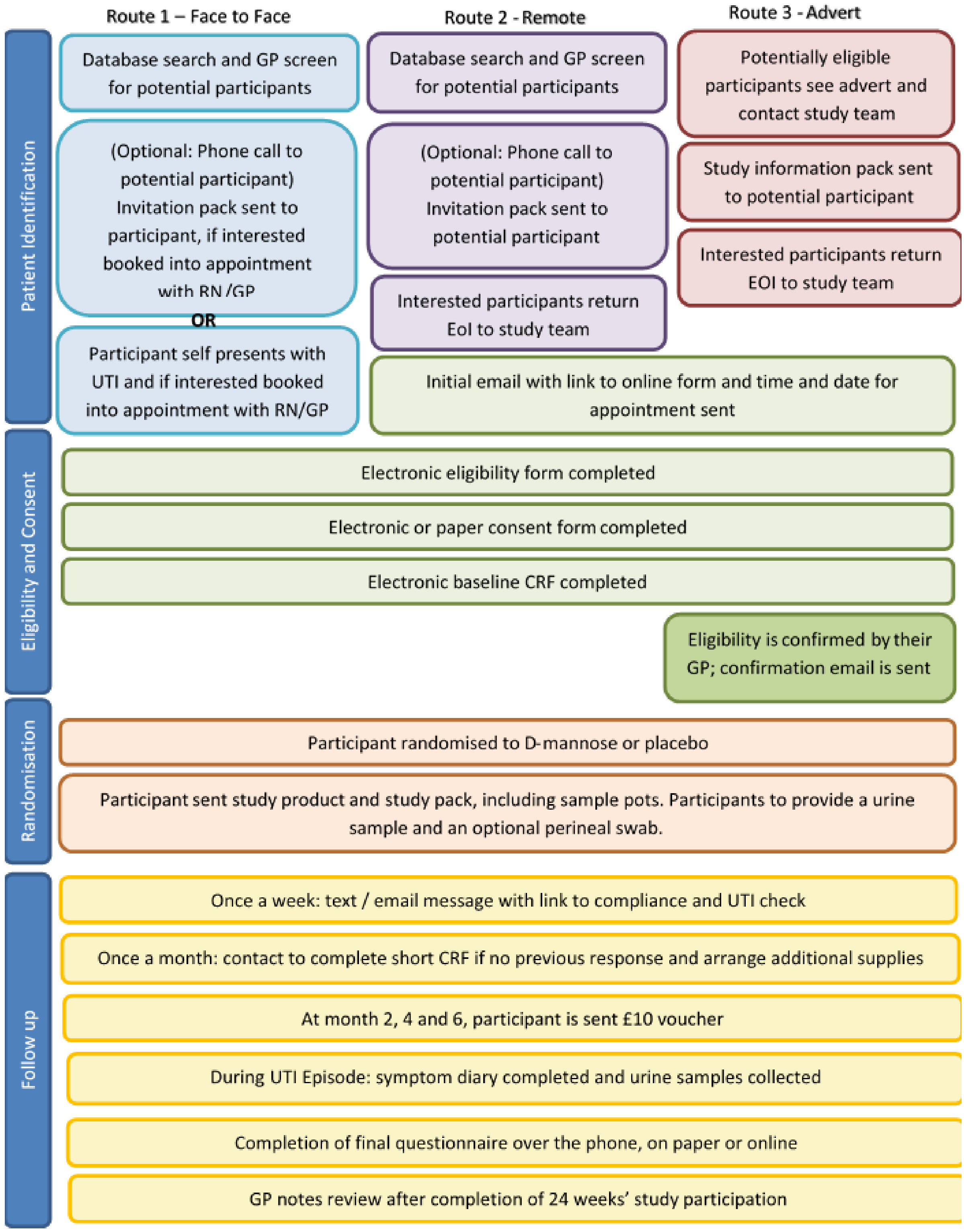

APPENDIX III. SCHEDULE OF PROCEDURES

Procedures	Participant Contacts									
	For those in route 1 and 2; mail out group	Telephone call 1*	Telephone call 2* or at GP practice	After telephone call 2 or baseline visit at GP practice	Weekly contact	Monthly contact	2 month contact	4 month contact	6 month contact	During UTI episode
Eligibility check by study team		✓								
Eligibility check by GP	✓			✓†						
Informed consent			✓							
Demographics		✓	✓							
Medical history		✓	✓							
Randomisation				✓						
Dispensing of study product				✓			✓	✓		
Compliance					✓	✓				
Laboratory tests				✓						✓
SAE assessment										✓
Symptom diary										✓
£10 voucher given							✓	✓	✓	

*Telephone call 2 can take place at the same time as phone call one if participant completed consent form has been received
†For those in route 3 recruitment group (recruitment via advert)

APPENDIX IV. FLOW DIAGRAM OF TRIAL PARTICIPANTS

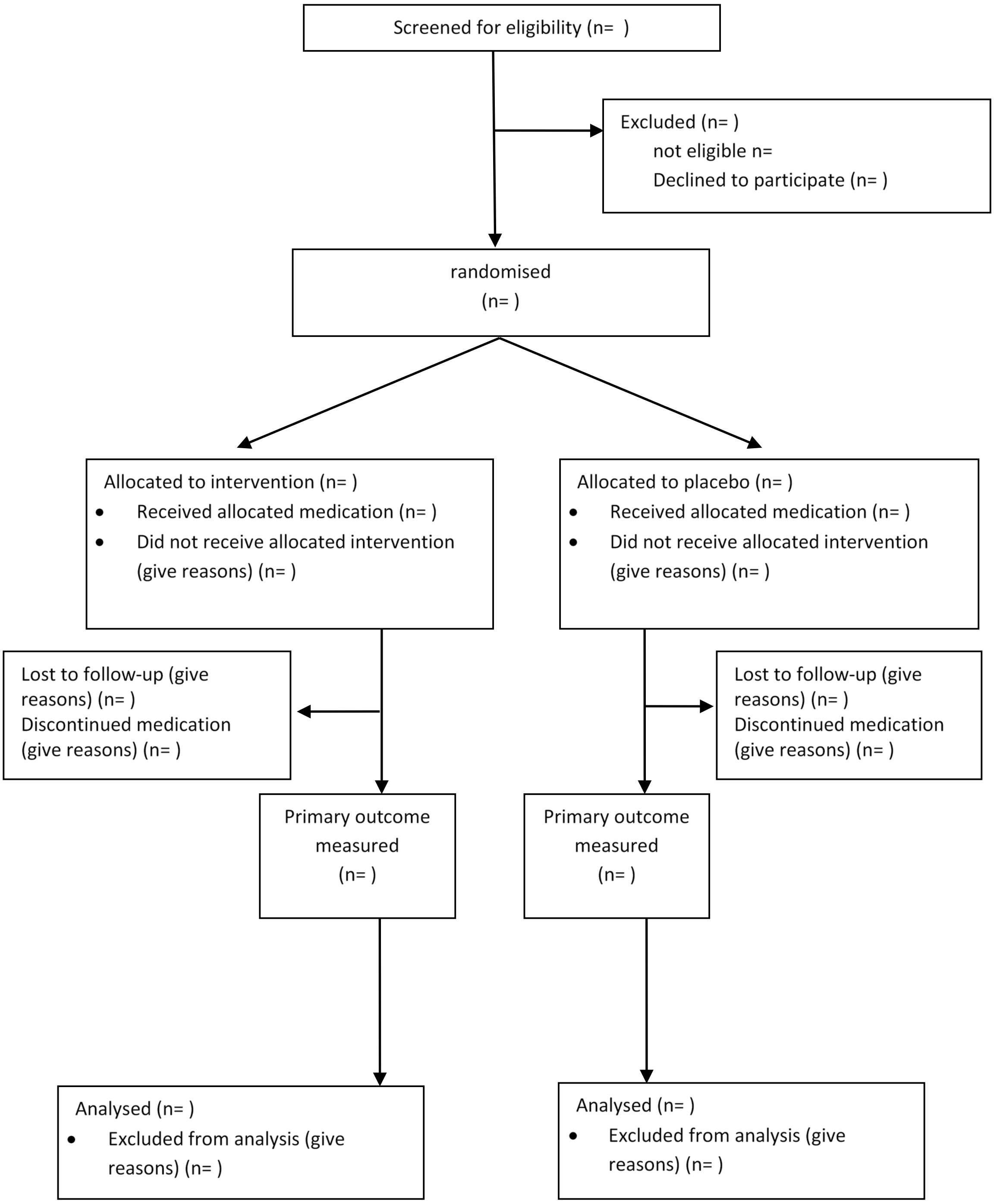

Supplement: Supplement 2. — Statistical Analysis Plan [file jamainternmed-e240264-s002.pdf]
