# Supplementary material for: d-Mannose for Prevention of Recurrent Urinary Tract Infection Among Women: A Randomized Clinical Trial
Source: JAMA Intern Med. 2024 Apr 8;184(6):619–28. doi: 10.1001/jamainternmed.2024.0264 (PMC11002776; doi:10.1001/jamainternmed.2024.0264)
Supplement: Supplement 3. — eTable 1. Additional Baseline Characteristics eTable 2. Summary statistics for the sensitivity analyses and the treatment difference between the randomised groups eTable 3. Summary statistics for the subgroup analyses and the treatment difference between the randomised groups eTable 4. Results from the complier-average causal effect (CACE) analysis eTable 5. Details of clinically reported UTIs, urine cultures requested and microbiologically confirmed UTIs by randomized group eTable 6. Duration of symptoms and antibiotic use in days, and number of clinically suspected UTIs by study arm eTable 7. Post Hoc exploratory analysis comparing the number of days antibiotic use, excluding women with a number of days of antibiotics prescription over 3 standard deviations from the mean eTable 8. Mean number of courses and Mean Defined Daily Dosage of antibiotics consumed split by type [file jamainternmed-e240264-s003.pdf]

Supplemental Online Content

Hayward G, Mort S, Hay AD, et al. D-Mannose to for prevention of recurrent urinary tract infection among women: a randomized clinical trial. *JAMA Intern Med*. Published online April 8, 2024. doi:10.1001/jamainternmed.2024.0264

eTable 1. Additional Baseline Characteristics

eTable 2. Summary statistics for the sensitivity analyses and the treatment difference between the randomised groups

eTable 3. Summary statistics for the subgroup analyses and the treatment difference between the randomised groups

eTable 4. Results from the complier-average causal effect (CACE) analysis

eTable 5. Details of clinically reported UTIs, urine cultures requested and microbiologically confirmed UTIs by randomized group

eTable 6. Duration of symptoms and antibiotic use in days, and number of clinically suspected UTIs by study arm

eTable 7. Post Hoc exploratory analysis comparing the number of days antibiotic use, excluding women with a number of days of antibiotics prescription over 3 standard deviations from the mean

eTable 8. Mean number of courses and Mean Defined Daily Dosage of antibiotics consumed split by type

This supplemental material has been provided by the authors to give readers additional information about their work.

© 2024 Hayward G et al. *JAMA Internal Medicine.*

eTable 1. Additional Baseline Characteristics

	D-mannose (N=303)	Placebo (N=295)	Overall (N=598)
**Severity of fever for previous UTI episodes[a]**			
Mean (SD)	1.2 (1.29)	1.3 (1.46)	1.3 (1.38)
Median (IQR)	1.0 (0.0 to 2.0)	1.0 (0.0 to 2.3)	1.0 (0.0 to 2.0)
[Range]	[0.0 to 5.0]	[0.0 to 6.0]	[0.0 to 6.0]
Missing	*19*	*16*	*35*
**Severity of burning or pain when passing urine for previous UTI episodes[a]**			
Mean (SD)	3.2 (1.61)	3.3 (1.71)	3.2 (1.66)
Median (IQR)	3.0 (2.0 to 4.3)	3.7 (2.0 to 4.7)	3.3 (2.0 to 4.3)
[Range]	[0.0 to 6.0]	[0.0 to 6.0]	[0.0 to 6.0]
Missing	*9*	*9*	*18*
**Severity of urgency (having to go in a hurry) for previous UTI episodes[a]**			
Mean (SD)	3.5 (1.40)	3.6 (1.39)	3.5 (1.40)
Median (IQR)	3.5 (2.7 to 4.7)	4.0 (3.0 to 4.7)	3.7 (3.0 to 4.7)
[Range]	[0.0 to 6.0]	[0.0 to 6.0]	[0.0 to 6.0]
Missing	*9*	*9*	*18*
**Severity of day time frequency (having to go more often than usual in the day) for previous UTI episodes[a]**			
Mean (SD)	3.5 (1.26)	3.5 (1.30)	3.5 (1.28)
Median (IQR)	3.3 (3.0 to 4.3)	3.7 (2.7 to 4.5)	3.4 (2.7 to 4.3)
[Range]	[0.0 to 6.0]	[0.0 to 6.0]	[0.0 to 6.0]
Missing	*8*	*10*	*18*
**Severity of night time frequency (going more often than usual at night) for previous UTI episodes[a]**			
Mean (SD)	2.9 (1.46)	2.9 (1.41)	2.9 (1.43)
Median (IQR)	3.0 (2.0 to 4.0)	3.0 (2.0 to 4.0)	3.0 (2.0 to 4.0)
[Range]	[0.0 to 6.0]	[0.0 to 6.0]	[0.0 to 6.0]
Missing	*8*	*9*	*17*
**Severity of inability to do usual activities for previous UTI episodes[a]**			
Mean (SD)	2.1 (1.62)	2.3 (1.63)	2.2 (1.63)
Median (IQR)	2.0 (1.0 to 3.3)	2.0 (1.0 to 3.3)	2.0 (1.0 to 3.3)
[Range]	[0.0 to 6.0]	[0.0 to 6.0]	[0.0 to 6.0]
Missing	*13*	*11*	*24*
**Severity of feeling generally unwell for previous UTI episodes[a]**			
Mean (SD)	2.6 (1.45)	2.7 (1.47)	2.7 (1.46)
Median (IQR)	2.7 (1.7 to 3.8)	3.0 (1.7 to 3.7)	3.0 (1.7 to 3.7)
[Range]	[0.0 to 6.0]	[0.0 to 6.0]	[0.0 to 6.0]
Missing	*7*	*8*	*15*
**Severity of nausea for previous UTI episodes[a]**			
Mean (SD)	1.0 (1.39)	1.0 (1.43)	1.0 (1.41)
Median (IQR)	0.0 (0.0 to 1.7)	0.0 (0.0 to 2.0)	0.0 (0.0 to 2.0)
[Range]	[0.0 to 6.0]	[0.0 to 6.0]	[0.0 to 6.0]
Missing	*15*	*12*	*27*
**Severity of loin pain for previous UTI episodes[a]**			

© 2024 Hayward G et al. *JAMA Internal Medicine.*

	D-mannose (N=303)	Placebo (N=295)	Overall (N=598)
Mean (SD)	2.1 (1.70)	2.2 (1.73)	2.1 (1.72)
Median (IQR)	2.0 (0.3 to 3.3)	2.0 (0.3 to 3.7)	2.0 (0.3 to 3.3)
[Range]	[0.0 to 6.0]	[0.0 to 6.0]	[0.0 to 6.0]
Missing	*12*	*9*	*21*
**Severity of fever for current UTI episode**			
Normal / not affected, n(%)	28 (44.4%)	51 (68.9%)	79 (57.7%)
Very little problem, n(%)	14 (22.2%)	9 (12.2%)	23 (16.8%)
Slight problem, n(%)	13 (20.6%)	9 (12.2%)	22 (16.1%)
Moderately bad, n(%)	4 (6.3%)	1 (1.4%)	5 (3.6%)
Bad, n(%)	3 (4.8%)	1 (1.4%)	4 (2.9%)
Very bad, n(%)	1 (1.6%)	2 (2.7%)	3 (2.2%)
As bad as it could be, n(%)	0 (0.0%)	1 (1.4%)	1 (0.7%)
Missing	*4*	*1*	*5*
**Severity of burning or pain when passing urine for current UTI episode**			
Normal / not affected, n(%)	10 (15.2%)	17 (23.0%)	27 (19.3%)
Very little problem, n(%)	11 (16.7%)	7 (9.5%)	18 (12.9%)
Slight problem, n(%)	20 (30.3%)	14 (18.9%)	34 (24.3%)
Moderately bad, n(%)	14 (21.2%)	15 (20.3%)	29 (20.7%)
Bad, n(%)	8 (12.1%)	13 (17.6%)	21 (15.0%)
Very bad, n(%)	2 (3.0%)	5 (6.8%)	7 (5.0%)
As bad as it could be, n(%)	1 (1.5%)	3 (4.1%)	4 (2.9%)
Missing	*1*	*1*	*2*
**Severity of urgency (having to go in a hurry) for current UTI episode**			
Normal / not affected, n(%)	6 (9.7%)	6 (8.2%)	12 (8.9%)
Very little problem, n(%)	8 (12.9%)	3 (4.1%)	11 (8.1%)
Slight problem, n(%)	19 (30.6%)	26 (35.6%)	45 (33.3%)
Moderately bad, n(%)	14 (22.6%)	15 (20.5%)	29 (21.5%)
Bad, n(%)	6 (9.7%)	9 (12.3%)	15 (11.1%)
Very bad, n(%)	5 (8.1%)	11 (15.1%)	16 (11.9%)
As bad as it could be, n(%)	4 (6.5%)	3 (4.1%)	7 (5.2%)
Missing	*5*	*2*	*7*
**Severity of day time frequency (having to go more often than usual in the day) for current UTI episode**			
Normal / not affected, n(%)	7 (10.8%)	6 (8.1%)	13 (9.4%)
Very little problem, n(%)	6 (9.2%)	2 (2.7%)	8 (5.8%)
Slight problem, n(%)	24 (36.9%)	25 (33.8%)	49 (35.3%)
Moderately bad, n(%)	17 (26.2%)	17 (23.0%)	34 (24.5%)
Bad, n(%)	4 (6.2%)	11 (14.9%)	15 (10.8%)
Very bad, n(%)	3 (4.6%)	11 (14.9%)	14 (10.1%)
As bad as it could be, n(%)	4 (6.2%)	2 (2.7%)	6 (4.3%)
Missing	*2*	*1*	*3*
**Severity of night time frequency (going more often than usual at night) for current UTI episode**			
Normal / not affected, n(%)	9 (13.8%)	9 (12.2%)	18 (12.9%)
Very little problem, n(%)	10 (15.4%)	12 (16.2%)	22 (15.8%)
Slight problem, n(%)	21 (32.3%)	20 (27.0%)	41 (29.5%)
Moderately bad, n(%)	12 (18.5%)	14 (18.9%)	26 (18.7%)
Bad, n(%)	10 (15.4%)	8 (10.8%)	18 (12.9%)
Very bad, n(%)	1 (1.5%)	9 (12.2%)	10 (7.2%)
As bad as it could be, n(%)	2 (3.1%)	2 (2.7%)	4 (2.9%)

© 2024 Hayward G et al. *JAMA Internal Medicine.*

	D-mannose (N=303)	Placebo (N=295)	Overall (N=598)
Missing	*2*	*1*	*3*
**Severity of inability to do usual activities for current UTI episode**			
Normal / not affected, n(%)	25 (39.7%)	26 (35.1%)	51 (37.2%)
Very little problem, n(%)	11 (17.5%)	10 (13.5%)	21 (15.3%)
Slight problem, n(%)	16 (25.4%)	24 (32.4%)	40 (29.2%)
Moderately bad, n(%)	3 (4.8%)	6 (8.1%)	9 (6.6%)
Bad, n(%)	3 (4.8%)	5 (6.8%)	8 (5.8%)
Very bad, n(%)	4 (6.3%)	2 (2.7%)	6 (4.4%)
As bad as it could be, n(%)	1 (1.6%)	1 (1.4%)	2 (1.5%)
Missing	*4*	*1*	*5*
**Severity of feeling generally unwell for current UTI episode**			
Normal / not affected, n(%)	14 (21.9%)	21 (28.4%)	35 (25.4%)
Very little problem, n(%)	11 (17.2%)	14 (18.9%)	25 (18.1%)
Slight problem, n(%)	17 (26.6%)	17 (23.0%)	34 (24.6%)
Moderately bad, n(%)	13 (20.3%)	12 (16.2%)	25 (18.1%)
Bad, n(%)	4 (6.3%)	7 (9.5%)	11 (8.0%)
Very bad, n(%)	3 (4.7%)	2 (2.7%)	5 (3.6%)
As bad as it could be, n(%)	2 (3.1%)	1 (1.4%)	3 (2.2%)
Missing	*3*	*1*	*4*
**Severity of nausea for current UTI episode**			
Normal / not affected, n(%)	41 (65.1%)	50 (67.6%)	91 (66.4%)
Very little problem, n(%)	7 (11.1%)	9 (12.2%)	16 (11.7%)
Slight problem, n(%)	7 (11.1%)	6 (8.1%)	13 (9.5%)
Moderately bad, n(%)	4 (6.3%)	6 (8.1%)	10 (7.3%)
Bad, n(%)	2 (3.2%)	1 (1.4%)	3 (2.2%)
Very bad, n(%)	1 (1.6%)	1 (1.4%)	2 (1.5%)
As bad as it could be, n(%)	1 (1.6%)	1 (1.4%)	2 (1.5%)
Missing	*4*	*1*	*5*
**Severity of loin pain for current UTI episode**			
Normal / not affected, n(%)	19 (30.2%)	24 (32.9%)	43 (31.6%)
Very little problem, n(%)	13 (20.6%)	12 (16.4%)	25 (18.4%)
Slight problem, n(%)	14 (22.2%)	12 (16.4%)	26 (19.1%)
Moderately bad, n(%)	9 (14.3%)	10 (13.7%)	19 (14.0%)
Bad, n(%)	4 (6.3%)	8 (11.0%)	12 (8.8%)
Very bad, n(%)	1 (1.6%)	6 (8.2%)	7 (5.1%)
As bad as it could be, n(%)	3 (4.8%)	1 (1.4%)	4 (2.9%)
Missing	*4*	*2*	*6*
**EQ-5D – Mobility**			
No problems in walking about, n(%)	223 (73.6%)	219 (74.2%)	442 (73.9%)
Slight problems, n(%)	44 (14.5%)	35 (11.9%)	79 (13.2%)
Moderate problems, n(%)	20 (6.6%)	29 (9.8%)	49 (8.2%)
Severe problems, n(%)	11 (3.6%)	10 (3.4%)	21 (3.5%)
Unable to walk about, n(%)	5 (1.7%)	2 (0.7%)	7 (1.2%)
Missing	*0*	*0*	*0*
**EQ-5D – Self-care**			
No problems washing or dressing, n(%)	284 (93.7%)	272 (92.2%)	556 (93.0%)
Slight problems, n(%)	12 (4.0%)	13 (4.4%)	25 (4.2%)
Moderate problems, n(%)	4 (1.3%)	5 (1.7%)	9 (1.5%)
Severe problems, n(%)	1 (0.3%)	4 (1.4%)	5 (0.8%)
Unable to wash or dress myself, n(%)	2 (0.7%)	1 (0.3%)	3 (0.5%)
Missing	*0*	*0*	*0*

© 2024 Hayward G et al. *JAMA Internal Medicine.*

	D-mannose (N=303)	Placebo (N=295)	Overall (N=598)
**EQ-5D – Usual activities**			
No problems doing usual activities, n(%)	238 (78.5%)	211 (71.5%)	449 (75.1%)
Slight problems, n(%)	36 (11.9%)	48 (16.3%)	84 (14.0%)
Moderate problems, n(%)	23 (7.6%)	24 (8.1%)	47 (7.9%)
Severe problems, n(%)	4 (1.3%)	9 (3.1%)	13 (2.2%)
Unable to do usual activities, n(%)	2 (0.7%)	3 (1.0%)	5 (0.8%)
Missing	*0*	*0*	*0*
**EQ-5D – Pain/discomfort**			
No pain or discomfort, n(%)	149 (49.2%)	137 (46.4%)	286 (47.8%)
Slight pain or discomfort, n(%)	90 (29.7%)	84 (28.5%)	174 (29.1%)
Moderate pain or discomfort, n(%)	49 (16.2%)	60 (20.3%)	109 (18.2%)
Severe pain or discomfort, n(%)	10 (3.3%)	10 (3.4%)	20 (3.3%)
Extreme pain or discomfort, n(%)	5 (1.7%)	4 (1.4%)	9 (1.5%)
Missing	*0*	*0*	*0*
**EQ-5D – Anxiety/depression**			
I am not anxious or depressed, n(%)	196 (64.7%)	191 (64.7%)	387 (64.7%)
Slightly anxious or depressed, n(%)	73 (24.1%)	70 (23.7%)	143 (23.9%)
Moderately anxious or depressed, n(%)	29 (9.6%)	26 (8.8%)	55 (9.2%)
Severely anxious or depressed, n(%)	4 (1.3%)	4 (1.4%)	8 (1.3%)
Extremely anxious or depressed, n(%)	1 (0.3%)	4 (1.4%)	5 (0.8%)
Missing	*0*	*0*	*0*
**EQ-5D index score**			
Mean (SD)	0.8 (0.21)	0.8 (0.21)	0.8 (0.21)
Median (IQR)	0.8 (0.7 to 1.0)	0.8 (0.7 to 1.0)	0.8 (0.7 to 1.0)
[Range]	[-0.2 to 1.0]	[-0.3 to 1.0]	[-0.3 to 1.0]
Missing	*0*	*0*	*0*
**EQ-5D VAS**			
Mean (SD)	76.6 (18.72)	76.1 (18.91)	76.3 (18.80)
Median (IQR)	80.0 (70.0 to 90.0)	80.0 (70.0 to 90.0)	80.0 (70.0 to 90.0)
[Range]	[10.0 to 100.0]	[3.0 to 100.0]	[3.0 to 100.0]
Missing	*0*	*0*	*0*

[a]For the previous three UTI episodes

eTable 2. Summary statistics for the sensitivity analyses and the treatment difference between the randomised groups

	D-mannose (N=303)	Placebo (N=295)	Treatment difference [95% CI]	P-value
**Per-protocol analysis (at least 4 days / 20 weeks consumption)**	161	146		
Adjusted relative risk [95% CI][a]			1.01 [0.84 to 1.21]	0.94
Yes, n(%)	80 (50.0%)	72 (49.7%)		
No, n(%)	80 (50.0%)	73 (50.3%)		
Missing	*1*	*1*		
**Per-protocol analysis (at least 3 days / 15 weeks)**	214	208		
Adjusted relative risk [95% CI][a]			0.97 [0.82 to 1.15]	0.70
Yes, n(%)	109 (51.4%)	110 (53.1%)		
No, n(%)	103 (48.6%)	97 (46.9%)		

© 2024 Hayward G et al. *JAMA Internal Medicine.*

	D-mannose (N=303)	Placebo (N=295)	Treatment difference [95% CI]	P-value
Missing	*2*	*1*		
**Factors that predict missingness**				
Adjusted relative risk [95% CI][b]			0.92 [0.79 to 1.06]	0.23
Yes, n(%)	150 (51.0%)	161 (55.7%)		
No, n(%)	144 (49.0%)	128 (44.3%)		
Missing	*9*	*6*		
**Imputing missing primary outcome as 'yes' (experienced at least one further episode of a clinically suspected UTI)**				
Adjusted relative risk [95% CI][a]			0.93 [0.81 to 1.06]	0.28
Yes, n(%)	159 (52.5%)	167 (56.6%)		
No, n(%)	144 (47.5%)	128 (43.4%)		
Missing	*0*	*0*		
**Imputing missing primary outcome as 'no' (did not experience at least one further episode of a clinically suspected UTI)**				
Adjusted relative risk [95% CI][a]			0.91 [0.79 to 1.05]	0.18
Yes, n(%)	150 (49.5%)	161 (54.6%)		
No, n(%)	153 (50.5%)	134 (45.4%)		
Missing	*0*	*0*		

D-mannose versus placebo. [a]Log-Poisson generalised linear mixed effects model with robust standard errors modelled against randomised arm as a fixed effect; and site as a random effect. [b]Log-Poisson generalised linear mixed model with robust standard errors modelled against randomised arm, and did you take anything to help with your symptoms for previous UTI episodes as fixed effects; and site as a random effect

© 2024 Hayward G et al. *JAMA Internal Medicine.*

eTable 3. Summary statistics for the subgroup analyses and the treatment difference between the randomised groups

	D-mannose (N=303)	Placebo (N=295)	Treatment difference Adjusted RR [95% CI][a]	Test of Interaction (P-value)
**Frequency of UTIs at baseline**				0.58
**≤Median number of UTIs at baseline**			0.97 [0.80 to 1.18]	
N	191	169		
Primary outcome – Yes, n(%)	87 (46.5%)	80 (47.9%)		
Primary outcome – No, n(%)	100 (53.5%)	87 (52.1%)		
Missing	*4*	*2*		
**>Median number of UTIs at baseline**			0.91 [0.75 to 1.09]	
N	108	123		
Primary outcome – Yes, n(%)	62 (60.2%)	79 (66.4%)		
Primary outcome – No, n(%)	41 (39.8%)	40 (33.6%)		
Missing	*5*	*4*		
**Menopause**				0.12
**Pre-menopausal**			0.74 [0.54 to 1.01]	
N	86	97		
Primary outcome – Yes, n(%)	34 (40.0%)	51 (54.3%)		
Primary outcome – No, n(%)	51 (60.0%)	43 (45.7%)		
Missing	*1*	*3*		
**Post-menopausal**			0.98 [0.84 to 1.15]	
N	194	182		
Primary outcome – Yes, n(%)	105 (56.5%)	103 (57.5%)		
Primary outcome – No, n(%)	81 (43.5%)	76 (42.5%)		
Missing	*8*	*3*		

D-mannose versus placebo. [a]Log-Poisson generalised linear mixed effects model with robust standard errors of the proportion of women experiencing at least one further episode of a clinically suspected UTI modelled against randomised arm, an indicator variable for the subgroup, and an interaction between randomised arm and the subgroup indicator variable as fixed effects; and site as a random effect. Level of significance = 0.05

eTable 4. Results from the complier-average causal effect (CACE) analysis

	D-mannose (N=303)	Placebo (N=295)
**CACE analysis for the D-Mannose treatment (4 days / 20 weeks)**		
Complied, n/N (%)	161/303 (53.1)	0/295 (0.0)
Did not comply, n/N (%)	142/303 (46.9)	295/295 (100.0)
Unadjusted effect[†]	-0.22 [-0.60 to 0.16]; P=0.26	
Adjusted effect[a†]	-0.20 [-0.60 to 0.21]; P=0.34	
**CACE analysis for the D-Mannose treatment (3 days / 15 weeks)**		
Complied, n/N (%)	214/303 (70.6)	0/295 (0.0)
Did not comply, n/N (%)	89/303 (29.4)	295/295 (100.0)
Unadjusted effect[†]	-0.16 [-0.45 to 0.12]; P=0.26	
Adjusted effect[b†]	-0.16 [-0.45 to 0.14]; P=0.30	

D-mannose versus placebo. [a]Adjusted for factors found to be predictive of compliance (age, severity of fever for previous UTI episodes, severity of feeling generally unwell for previous UTI episodes, paracetamol use to help with symptoms for previous UTI episodes, ibuprofen use to help with symptoms for previous UTI episodes, cranberry juice use to help with symptoms for previous UTI episodes, EQ5D VAS). [b]Adjusted for factors found to be predictive of compliance (age, severity of fever for previous UTI episodes, severity of nausea for previous UTI episodes, cranberry juice use to help with symptoms for previous UTI episodes, other medication use to help with symptoms for previous UTI episodes). †Level of significance = 0.05.

eTable 5. Details of clinically reported UTIs, urine cultures requested and microbiologically confirmed UTIs by randomized group.[a]

	D-mannose	Placebo
At least one clinically suspected UTI for which participants contacted ambulatory care	150/294 (51.0)	161/289 (55.7)
Total number of clinically suspected UTIs recorded in the clinical record [b]	270	303
Number of recorded UTIs with at least one urine culture	166/270 (61.5)	191/303 (63.0)
Number of microbiologically confirmed UTIs [bc]	92/166 (55.4)	100/191 (52.4)
At least one Microbiologically confirmed UTI (per individual) following contact with healthcare for UTI	60/294 (20.4)	68/289 (23.5)

[a]post-hoc exploratory analysis requested by peer review [b]including multiple UTIs per person [c]Colony counts of 10^4-10^5 or >10^5 cfu/ml

eTable 6. Duration of symptoms and antibiotic use in days, and number of clinically suspected UTIs by study arm

	D-mannose (N=303)	Placebo (N=295)	Overall (N=598)
Duration of symptoms (days)			
0	136 (56.7%)	130 (56.8%)	266 (56.7%)
1	34 (14.2%)	29 (12.7%)	63 (13.4%)
2	13 (5.4%)	19 (8.3%)	32 (6.8%)
3	17 (7.1%)	10 (4.4%)	27 (5.8%)
4	11 (4.6%)	9 (3.9%)	20 (4.3%)
5	7 (2.9%)	3 (1.3%)	10 (2.1%)
>5	22 (2.9%)	29 (12.7%)	51 (10.9%)
Missing	*63*	*66*	*129*
Days of antibiotic use			
0	154 (52.4%)	143 (49.5%)	297 (50.8%)
1-7	75 (25.5%)	74 (25.6%)	149 (25.5%)
8-14	32 (10.9%)	34 (11.8%)	66 (11.3%)
15-21	22 (7.5%)	21 (7.3%)	43 (7.4%)
22-28	2 (0.7%)	3 (1.0%)	5 (0.9%)
29-35	2 (0.7%)	4 (1.4%)	6 (1.0%)
36-42	3 (1.0%)	4 (1.4%)	7 (1.2%)
43-49	2 (0.7%)	4 (1.4%)	6 (1.0%)
50-56	0 (0.0%)	1 (0.3%)	1 (0.2%)
57-63	1 (0.3%)	0 (0.0%)	1 (0.2%)
64-70	0 (0.0%)	1 (0.3%)	1 (0.2%)
>70 (=161)	1 (0.3%)	0 (0.0%)	1 (0.2%)

© 2024 Hayward G et al. *JAMA Internal Medicine.*

Missing	*9*	*6*	*13*
Number of clinically suspected UTIs			
0	144 (49.0%)	128 (44.3%)	272 (46.7%)
1	79 (26.9%)	74 (25.6%)	153 (26.2%)
2	40 (13.6%)	58 (20.1%)	98 (16.8%)
3	20 (6.8%)	15 (5.2%)	35 (6.0%)
4	5 (1.7%)	7 (2.4%)	12 (2.1%)
5	5 (1.7%)	4 (1.4%)	9 (1.5%)
>5	1 (0.3%)	3 (1.0%)	4 (0.7%)
Missing	*9*	*6*	*15*

eTable 7. Post Hoc exploratory analysis comparing the number of days antibiotic use, excluding women with a number of days of antibiotics prescription over 3 standard deviations from the mean

	**D-mannose (N=303)**	**Placebo (N=295)**	**Median Difference [95% CI]**	**P-value**
**Number of days prescribed antibiotics for UTI**				
Adjusted median difference [95% CI]			0.00 [-1.40 to 1.40]	>0.99
Mean (SD)	4.7 (7.09)	5.1 (7.46)		
Median (IQR)	0.0 (0.0 to 7.0)	0.0 (0.0 to 7.0)		
[Range]	[0.0 to 39.0]	[0.0 to 38.0]		
Missing	13	14		

D-mannose versus placebo. [a]Quantile regression modelled against randomised arm Level of significance: 0.05

eTable 8. Mean number of courses and Mean Defined Daily Dosage of antibiotics consumed split by type

	**D-Mannose (N=303)**	**Placebo (N=295)**
**Nitrofurantoin**		
N	109	107
Mean (SD) number of courses	1.6 (0.96)	1.5 (0.77)
Mean (SD) defined daily dose (DDD)	1.0 (0.13)	1.0 (0.07)
**Trimethoprim**		
N	28	34
Mean (SD) number of courses	1.5 (0.75)	1.3 (0.59)
Mean (SD) defined daily dose (DDD)	1.0 (0.00)	1.0 (0.00)
**Pivmecillinam**		
N	16	21
Mean (SD) number of courses	1.6 (1.21)	1.7 (1.02)
Mean (SD) defined daily dose (DDD)	0.9 (0.03)	0.9 (0.02)
**Ciprofloxacin**		
N	5	13

© 2024 Hayward G et al. *JAMA Internal Medicine.*

Mean (SD) number of courses	1.0 (0.00)	1.3 (0.85)
Mean (SD) defined daily dose (DDD)	1.0 (0.00)	1.0 (0.00)
**Co-amoxiclav**		
N	6	13
Mean (SD) number of courses	1.0 (0.00)	1.2 (0.55)
Mean (SD) defined daily dose (DDD)	1.3 (0.00)	1.2 (0.19)
**Amoxicillin**		
N	7	7
Mean (SD) number of courses	1.4 (0.79)	1.1 (0.38)
Mean (SD) defined daily dose (DDD)	1.0 (0.00)	0.9 (0.19)
**Cephalexin**		
N	3	6
Mean (SD) number of courses	1.0 (0.00)	1.7 (0.82)
Mean (SD) defined daily dose (DDD)	0.5 (0.00)	0.5 (0.00)

© 2024 Hayward G et al. *JAMA Internal Medicine.*
