# Supplementary material for: d-Mannose for Prevention of Recurrent Urinary Tract Infection Among Women: A Randomized Clinical Trial
Source: JAMA Intern Med. 2024 Apr 8;184(6):619–28. doi: 10.1001/jamainternmed.2024.0264 (PMC11002776; doi:10.1001/jamainternmed.2024.0264)
Supplement: Supplement 4. — Data Sharing Statement [file jamainternmed-e240264-s004.pdf]

Data Sharing Statement

Hayward. d-Mannose for Prevention of Recurrent Urinary Tract Infection Among Women. *JAMA Intern Med*. Published April 08, 2024. doi:10.1001/jamainternmed.2024.0264

Data
Data available: Yes
Data types: Deidentified participant data
How to access data: Requests for de-identified participant level data collected during this study should be made to the Nuffield Department of Primary Care hosted Datasets Independent Scientific Committee (PrimDISC): primdisc@phc.ox.ac.uk. Data will be released following review and approval by PrimDISC of a protocol, statistical analysis plan and the signing of a suitable data sharing agreement.
When available: With publication
